# β-glucan attenuates cognitive impairment via the gut-brain axis in diet-induced obese mice

**DOI:** 10.1186/s40168-020-00920-y

**Published:** 2020-10-02

**Authors:** Hongli Shi, Yinghua Yu, Danhong Lin, Peng Zheng, Peng Zhang, Minmin Hu, Qiao Wang, Wei Pan, Xiaoying Yang, Tao Hu, Qianqian Li, Renxian Tang, Feng Zhou, Kuiyang Zheng, Xu-Feng Huang

**Affiliations:** 1 Jiangsu Key Laboratory of Immunity and Metabolism, Department of Pathogen Biology and Immunology, Xuzhou Medical University, Xuzhou, 221004 Jiangsu China; 2 Illawarra Health and Medical Research Institute (IHMRI), University of Wollongong, Wollongong, NSW 2522 Australia; 3 School of Medicine, University of Wollongong, Wollongong, NSW 2522 Australia

**Keywords:** Cognition, Gut microbiota, Gut-brain axis, β-glucan, Obesity

## Abstract

**Background:**

“Western” style dietary patterns are characterized by a high proportion of highly processed foods rich in fat and low in fiber. This diet pattern is associated with a myriad of metabolic dysfunctions, including neuroinflammation and cognitive impairment. β-glucan, the major soluble fiber in oat and barley grains, is fermented in the lower gastrointestinal tract, potentially impacting the microbial ecosystem and thus may improve elements of cognition and brain function via the gut-brain axis. The present study aimed to evaluate the effect of β-glucan on the microbiota gut-brain axis and cognitive function in an obese mouse model induced by a high-fat and fiber-deficient diet (HFFD).

**Results:**

After long-term supplementation for 15 weeks, β-glucan prevented HFFD-induced cognitive impairment assessed behaviorally by object location, novel object recognition, and nesting building tests. In the hippocampus, β-glucan countered the HFFD-induced microglia activation and its engulfment of synaptic puncta, and upregulation of proinflammatory cytokine (TNF-α, IL-1β, and IL-6) mRNA expression. Also, in the hippocampus, β-glucan significantly promoted PTP1B-IRS-pAKT-pGSK3β-pTau signaling for synaptogenesis, improved the synaptic ultrastructure examined by transmission electron microscopy, and increased both pre- and postsynaptic protein levels compared to the HFFD-treated group. In the colon, β-glucan reversed HFFD-induced gut barrier dysfunction increased the thickness of colonic mucus (Alcian blue and mucin-2 glycoprotein immunofluorescence staining), increased the levels of tight junction proteins occludin and zonula occludens-1, and attenuated bacterial endotoxin translocation. The HFFD resulted in microbiota alteration, effects abrogated by long-term β-glucan supplementation, with the β-glucan effects on Bacteroidetes and its lower taxa particularly striking. Importantly, the study of short-term β-glucan supplementation for 7 days demonstrated pronounced, rapid differentiating microbiota changes before the cognitive improvement, suggesting the possible causality of gut microbiota profile on cognition. In support, broad-spectrum antibiotic intervention abrogated β-glucan’s effects on improving cognition, highlighting the role of gut microbiota to mediate cognitive behavior.

**Conclusion:**

This study provides the first evidence that β-glucan improves indices of cognition and brain function with major beneficial effects all along the gut microbiota-brain axis. Our data suggest that elevating consumption of β-glucan-rich foods is an easily implementable nutritional strategy to alleviate detrimental features of gut-brain dysregulation and prevent neurodegenerative diseases associated with Westernized dietary patterns.

Video Abstract

## Introduction

Neurodegenerative diseases, such as Alzheimer’s disease (AD) and related dementias, are a major contributor to morbidity, drastically impaired quality of life, and health care costs in an increasingly aging population [1]. These neurodegenerative diseases are not currently curable; however, the Lancet commission reported that more than one-third of dementia cases may be preventable through addressing lifestyle factors, including diet [1]. Increasing evidence showed that diets can influence the gut microbiome, potentially modulating brain functions and subsequent behavior, through the gut-brain axis. For example, high-fat diet-induced gut microbiota alterations can induce cognitive impairment in mice [2]. In addition, obese-type microbiota transplantation has been shown to disrupt the intestinal barrier and induce a cognitive decline in mice [3]. Furthermore, there is some evidence that microbial alteration is involved in neuroinflammation and cognitive impairment [3, 4], two important characteristics of AD pathogenesis and progression.

The gut microbiota profile serves as an important regulator for host intestinal homeostasis and the immune system. It is reported that *Ruminococcus* of Firmicutes phylum degraded mucus [5], while oral administration of the human commensal *Bacteroides fragilis* of Bacteroidetes phylum attenuated intestinal permeability in a mouse model of autism spectrum disorder [6]. Increased intestinal permeability allows hyper-translocation of bacterial lipopolysaccharide (LPS, endotoxin) into the blood circulation [2], which can trigger neuroinflammation. Systemic LPS administration activates microglia (principal immune cells in the central nervous system) and increases the expression of proinflammatory cytokines in the hippocampus of mice [7]. Proinflammatory cytokines, such as TNF-α, stimulate PTP1B transcription [8], which inhibits insulin signaling pIRS-pAKT-pGSK3β for synaptogenesis [9], indicating that PTP1B is an important mediator between neuroinflammation and synaptic impairment. Research, including from our lab, has shown that an obesogenic high-fat diet in rodents can lead to increased cytokine production and elevated PTP1B expression in the hippocampus [10, 11], an important brain region for cognition. It is known that AD patients often have certain degrees of systemic and neural inflammation, which may be associated with high-fat and fiber-deficiency diets [12]. Therefore, the dysregulation of the microbiota gut-brain axis may induce neuroinflammation, synaptic impairment, and subsequently cause cognitive decline.

Dietary fiber plays an important role in the proper functioning of the gut [13]. However, the diets of, for example, Americans, Australians, and Chinese have all experienced a significant decrease in fiber intake over time [14, 15]. Epidemiological studies have found that dietary fiber intake is positively associated with cognitive function [16, 17]. While the underlying mechanisms are still unclear, the gut-brain axis may play an important role. Soluble dietary fiber β-glucan is fermented in the lower gastrointestinal tract, potentially resulting in compositional shifts in gut microbiota, such as increased Bacteroidetes and decreased Firmicutes abundances at phylum level and increased *Bacteroides* and *Prevotella* but decreased *Dorea* at the genus level [18]. Gut microbiota has been recognized to contribute to host energy metabolism, immunity, or brain health via microbiota metabolites, such as LPS, short-chain fatty acids, amino acids, and vitamins [4, 19, 20]. The microbial-derived metabolites can be distributed well beyond the gastrointestinal tract and influence the physiology of the host. Gut microbiota disturbance can serve as a primary factor to augment LPS, proinflammatory cytokines, T helper cells, and monocytes, causing increased intestinal and blood-brain-barrier permeability. Altered gut microbiota has been associated with metabolic disorders and neurodegenerative diseases [3, 6]. The accumulative research data indicate that the manipulation of gut microbiota by enhancing the proportion of beneficial members of the community may be a strategy for the regulation of neurotransmitters and prevention or treatment of neurodegenerative diseases [21]. Therefore, β-glucan could have a strong beneficial effect on improving the gut microbiota-brain axis altered by Western diet (high-fat and fiber deficiency), which needs further investigation.

In this study, we used a chronic high-fat and fiber-deficient diet (HFFD), which induced a dysmetabolic cognitively impaired mouse model. Using this model, we assessed the effects of long-term β-glucan dietary supplementation on cognitive variables. Cognitive behavior tests and inflammation and insulin synapse signaling in the hippocampus, an important brain structure for cognition, were examined along with effects on gut microbiota, measures of colonic integrity (colonic mucus thickness and epithelial tight junction proteins), and endotoxemia (serum LPS). Furthermore, a short-term feeding study of β-glucan and, separately, an antibiotic intervention were used to assess the possibility of a causal relation between β-glucan-induced gut microbiota changes and effects on cognition.

## Results

### Long-term β-glucan supplementation ameliorated cognitive impairment in HFFD-fed mice

**Fig. 1:**
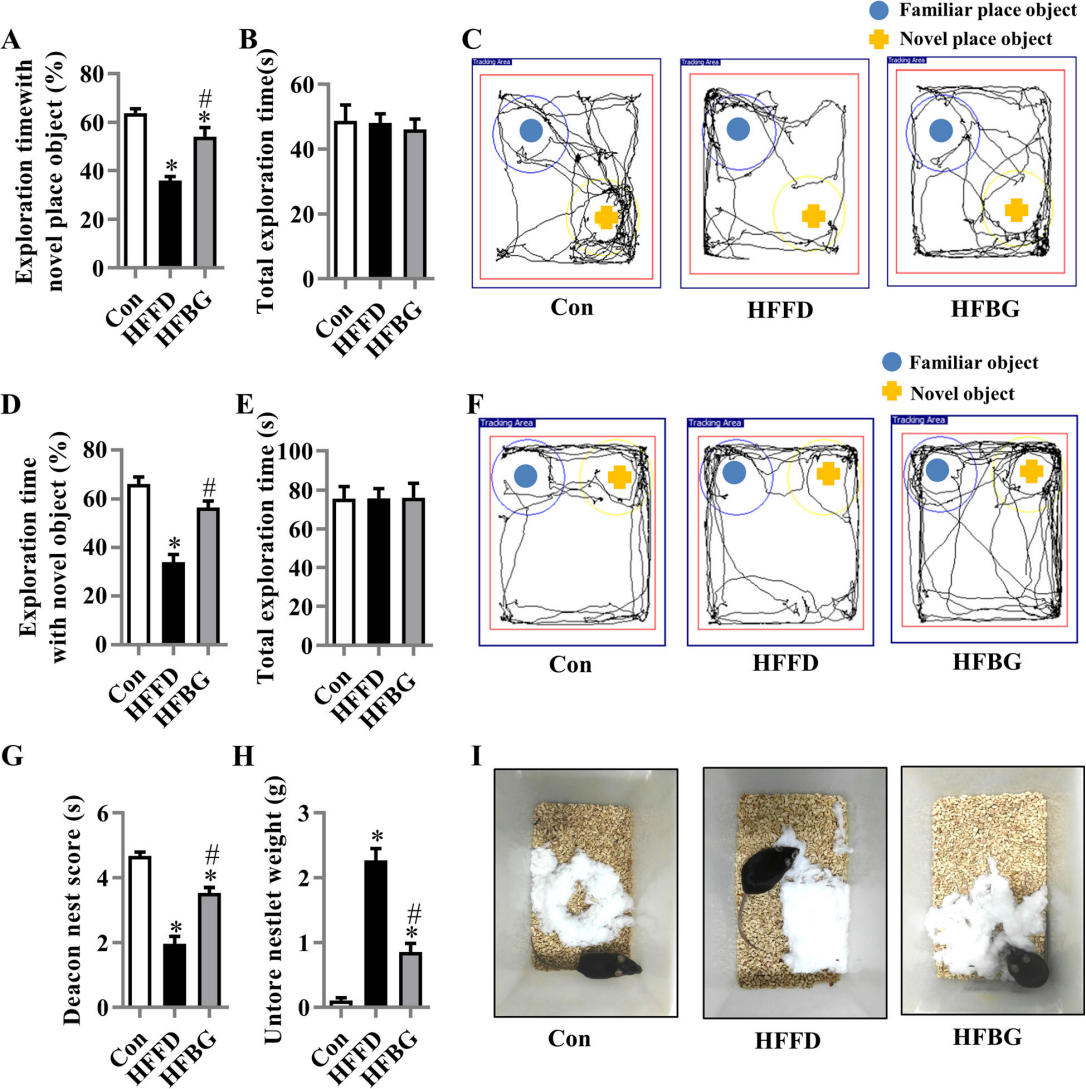
Long-term β-glucan supplementation ameliorated cognitive impairment in diet-induced obese mice. The object location test was performed to evaluate the spatial memory of the mice (**a**–**c**). **a** Percentage of time spent with the object in the novel place to total object exploration time. **b** The total object exploration time. **c** Representative track plots of control (Con), HFFD, and HFBG groups recorded by SMART video tracking system in the testing phase. Note that the control diet-fed mouse spent more time exploring the object in novel place whereas the HFFD mouse did not show preference to the object in a novel place. The novel object recognition test was performed to evaluate object recognition memory of the mice (**d**–**f**). **d** Percentage of time spent with the novel object to total object exploration time. **e** The total object exploration time. **f** Representative track plots of control (Con). The nest building test was used to assess the activity of daily living of mice (**g**–**i**). **g** The nest score and **h** untore nestler weight (amount of untore nesting material) (*n* = 15 mice). **i** Representative nest of control (Con), high-fat and fiber-deficient diet (HFFD), and β-glucan supplementation in HFFD (HFBG) groups. Values are mean ± SEM. ^*^p < 0.05 vs. Con. ^#^*p* < 0.05 vs. HFFD

To assess whether long-term β-glucan supplement could prevent HFFD-induced cognitive impairment, we performed object location, novel object recognition, and nesting behavioral tests, which explored hippocampus-dependent recognition memory and ability to perform activities of daily living [22–24]. In the object location test, after 15 weeks of supplementation, β-glucan significantly improved place recognition memory with increasing the place discrimination index (PDI), percentage of time spent with the object in a novel place in mice compared with the HFFD fed mice (*p* < 0.05) (Fig. 1a and c). The difference in PDI of the HFFD and β-glucan mice was not due to the variant general activity because both groups had similar total exploration time with the objects during the test phases (*p* > 0.05) (Fig. 1b). In the novel object recognition test, the novel object discrimination index (NODI) was significantly decreased in the HFFD group compared with the control and β-glucan supplementation group (Fig. 1d and f). The total exploration time of objects during the testing phase was comparable among the three groups (Fig. 1e). In the nesting behavioral test, the β-glucan group had higher deacon nest score (ability to build a nest) than that of the HFFD mice (*p* < 0.05) without a significant difference to control mice (Fig. 1d and f). In contrast, the untore nestlet weight (nest-building deficit) of β-glucan groups was significantly decreased compared with that of the HFFD group (Fig. 1e). However, the cognition index of the β-glucan group in object location and nesting behavior tests did not return to the level of the control group. Therefore, the supplementation of β-glucan attenuated the impairment of cognitive function induced by the HFFD.

### Long-term β-glucan supplementation suppressed the microglia activation and inflammation in the hippocampus of HFFD-fed mice

**Fig. 2:**
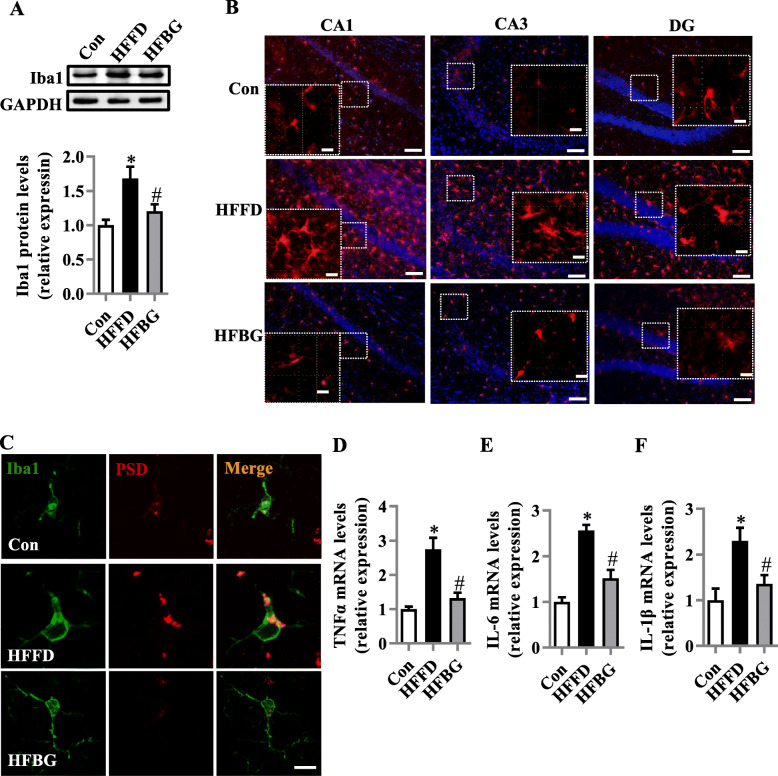
Long-term β-glucan supplementation suppressed the microglia activation and inflammation in the hippocampus of HFFD-induced obese mice. **a** The protein level of Iba1 in the hippocampus (*n* = 6). **b** The immunofluorescent staining of Iba1 in CA1, CA3, and DG of the hippocampus (*n* = 6) (scale bar 50 μm). The image captured from the box was marked with a dotted line (scale bar 10 μm). **c** The orthogonal view of the high-resolution confocal image shows the colocalization of Iba1 (green) and PSD95 (red) (scale bar 5 μm). **d**–**e** The mRNA expression of pro-inflammatory cytokines, TNFα, IL-1β, and IL-6 in the hippocampus (*n* = 5–6). Values are mean ± SEM. ^*^p < 0.05 vs. control (Con). ^#^*p* < 0.05 vs. high-fat and fiber-deficient (HFFD)

Activation of microglia is implicated in neuroinflammation and considered critical in the pathogenesis of neurodegenerative diseases [25, 26]. Western blot analysis showed β-glucan decreased Iba1 (activation marker of microglia) level compared with that of the HFFD group (*p* < 0.05, Fig. 2a). The morphology of microglia was further investigated by immunofluorescent staining with Iba1 antibody (Fig. 2b). In the HFFD group, the majority of cells showed the morphology of activated microglia with elongated soma and fewer branches along CA1, CA3, and DG of the hippocampus. In the control and β-glucan groups, the cells showed the characteristic of resting microglia consisting of a rod-shaped cell body with thin processes. We further determined the spatial location of microglia and synapses by double immunofluorescent staining of Iba-1 and PSD95 in the three groups (Fig. 2c). PSD95-positive puncta enveloped by microglia were increased in the HFFD group compared with control and β-glucan groups, suggesting that β-glucan attenuated the deleterious engulfment of synapses by activation of microglia seen in HFFD mice. Furthermore, β-glucan significantly prevented the upregulation of TNF-α, IL-1β, and IL-6 mRNA expression in the hippocampus (*p* < 0.05, Fig. 2d–f). These findings indicate that β-glucan prevented the HFFD-induced activation of microglia and neuroinflammation and associated deleterious engulfment of synapses.

### Long-term β-glucan supplementation improved PTP1B-IRS-pAKT-pGSK3β-pTau and synapse in the hippocampus of HFFD-fed mice

**Fig. 3:**
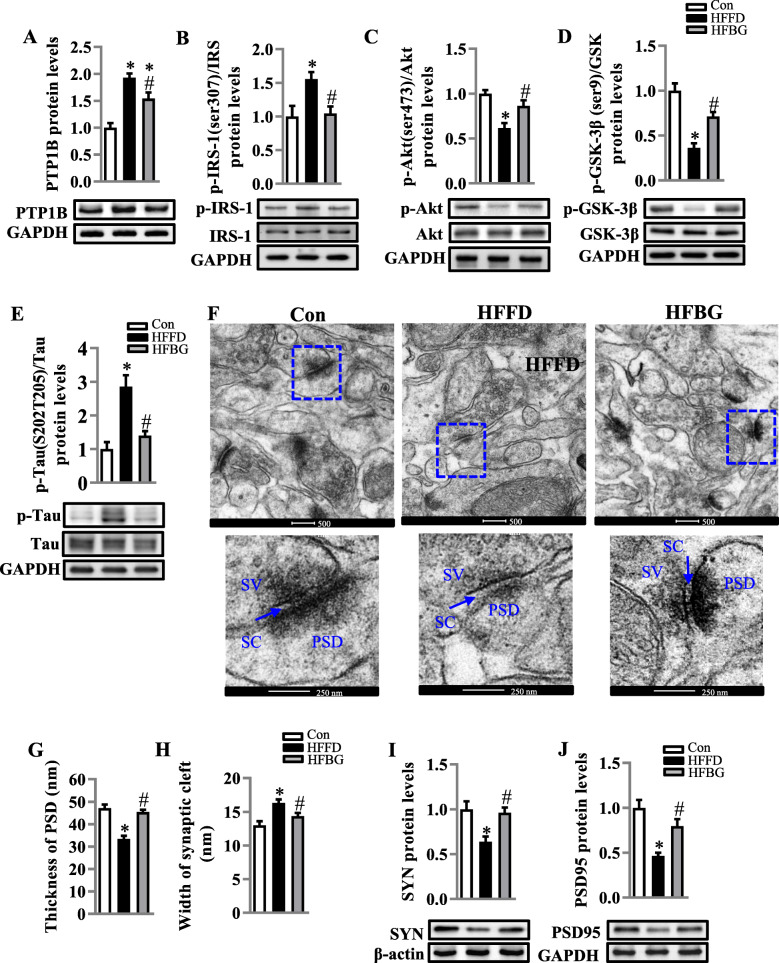
Long-term β-glucan supplementation improved PTP1B-IRS-pAKT-pGSK3β-pTau and synapse in the hippocampus of HFFD-induced obese mice. **a** The protein level of PTP1B in the hippocampus (*n* = 6). **b**–**d** Protein levels of p-IRS-1/IRS-1, p-Akt/Akt, and p-GSK3β/GSK3β (*n* = 6). **e** The protein level of p-Tau/Tau in the hippocampus (*n* = 4–6). **f** The ultrastructure of synapses on the electron micrograph in the hippocampus CA1 region of mice fed with different diets (scale bar 500 nm). The enlarged images of the second row were from the first row in the area indicated with a dotted line box (scale bar 250 nm). **g** and **h** Image analysis of the thickness of PSD and the width of the synaptic cleft (*n* = 3). PSD, postsynaptic density; SC, synaptic cleft; SV, synaptic vesicle. **i** and **j** The protein levels of SYN and PSD95. Values are mean ± SEM. ^*^p < 0.05 vs. control (Con). ^#^*p* < 0.05 vs. high-fat and fiber-deficient (HFFD)

PTP1B is an important mediator cross-linking inflammation and the disruption of insulin signaling IRS-pAKT-pGSK3β-pTau for synaptogenesis [8, 9]. Following the observation of β-glucan amelioration of neuroinflammation, we further evaluated the expression of PTP1B, pIRS, pAKT, pGSK3β, and pTau in the hippocampus. With the administration of β-glucan, PTP1B was significantly decreased compared to the HFFD group (*p* < 0.05), but still higher than the control group (*p* < 0.05) (Fig. 3a). β-glucan significantly inhibited the HFFD-induced increase of p-IRS-1 Ser307 (*p* < 0.05, Fig. 3b), which is associated with overexpression of PTP1B [27]. Consequently, the insulin signaling downstream molecules, p-Akt Ser473 and p-GSK3β Ser9, were downregulated in the HFFD group, while β-glucan ameliorated above alterations (all *p* < 0.05, Fig. 3c and d). Furthermore, β-glucan decreased the level of p-Tau (S202 + T205) compared with the HFFD group (*p* < 0.05, Fig. 3e). These results suggest that β-glucan inhibition of PTP1B may thereafter improve insulin signaling for synaptogenesis and inhibit Tau phosphorylation (a biomarker for Alzheimer’s pathology) in the hippocampus.

There is growing evidence that impaired insulin signaling and Tau over-phosphorylation contribute to synaptic degeneration [28, 29]. With transmission electron microscopy, the neuronal ultrastructure of synapses in the CA1 of the hippocampus was examined after long-term β-glucan consumption. In the HFFD group, presynaptic terminals were slightly swollen, while the thickness of the postsynaptic densities was decreased with a widening of synaptic clefts (Fig. 3f–h). In the β-glucan group, the synaptic structure was improved with thicker postsynaptic densities and narrower synaptic clefts compared with the HFFD group (both *p* < 0.05, Fig. 3g and h). Next, we measured two important presynaptic and postsynaptic proteins, synaptophysin (SYN) and postsynaptic density 95 (PSD95), by Western blot. Long-term β-glucan consumption significantly attenuated the decline of both SYN and PSD95 protein levels compared to the HFFD group (both *p* < 0.05, Fig. 3i and j).

### Long-term β-glucan supplementation prevented colonic mucosa barrier impairment and inflammation and ameliorated endotoxemia in HFFD-fed mice

**Fig. 4:**
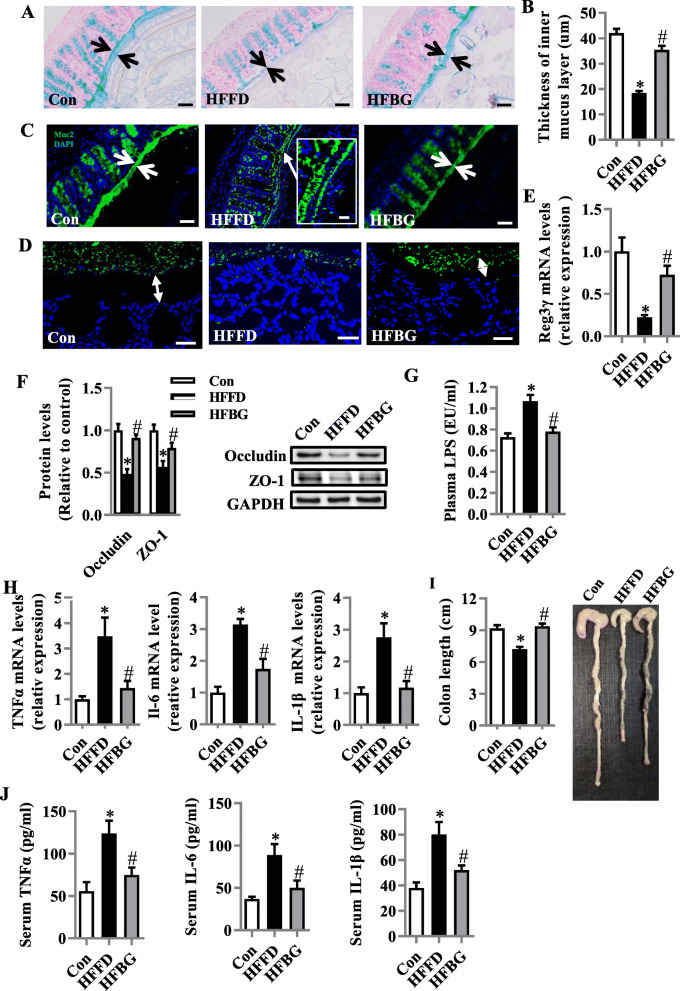
Long-term β-glucan supplementation prevented colonic mucosa barrier impairment and inflammation and ameliorated endotoxemia in HFFD-induced obese mice. **a** Alcian blue-stained colonic sections were showing the mucus layer (arrows). Opposing black arrows with shafts delineate the mucus layer that was measured (scale bar 50 μm). **b** The quantification of the colonic mucus layer was statistically analyzed (per section/2 sections per animal, *n* = 5). **c** Immunofluorescence images of colonic sections stained with Anti-MUC2 antibody and DAPI. Opposing white arrows with shafts delineate the mucus layer. Inset (HFFD group) shows a higher magnification of bacteria-sized, DAPI-stained particles in closer proximity to host epithelium and even crossing this barrier. Scale bar 50 μm, inset 10 μm. **d** FISH analysis of sections of the colon using the general bacterial probe EUB338-Alexa Fluor 488 (green), and nuclear staining DAPI (blue). Arrows indicate the distance between bacteria and epithelium. Scale bar 20 μm. **e** Quantitation of colonic Reg3γ by RT-PCR (*n* = 6). **f** Protein levels of occludin and ZO-1 in the colon (*n* = 5). **g** Serum endotoxin level (*n* = 10). **h** mRNA expression of TNF-α, IL-1β, and IL-6 in the colon (*n* = 5). **i** The quantification of colon length was statistically analyzed (*n* = 10) and representative images of colons. **j** TNF-α, IL-6, and IL-1β levels in the serum (*n* = 10). Values are mean ± SEM. ^*^p < 0.05 vs. control (Con). ^#^*p* < 0.05 vs. high-fat and fiber-deficient diet (HFFD)

Following β-glucan improving neuroinflammation and synaptic morphology, we examined the effects of β-glucan on intestinal barrier integrity. We found that β-glucan increased the thickness of colonic mucus compared with the HFFD group by using Alcian blue-staining (Fig. 4a and b) and mucin-2 glycoprotein (MUC2) immunofluorescence staining in the colon (Fig. 4c). MUC2, a disulfide cross-linked network, expands to form an inner layer which is rarely colonized by gut microbiota. Figure 4c (the insert) shows that in the HFFD group luminal bacteria were closer to the intestinal epithelium suggesting the degradation of the mucus layer by gut microbiota. Fluorescence in situ hybridization (FISH) was used to analyze the microbiota-epithelial localization in the colon (Fig. 4d). The distance of microbiota (green) to epithelial cell (blue) was shorter in the HFFD group, indicating microbiota encroachment, largely reversed by β-glucan with the FISH examination. The reduction of microbiota encroachment by β-glucan was accompanied by restoration of the expression of antimicrobial peptide Reg3γ (Fig. 4e), indicating β-glucan increases the ability for the mucosa to protect against bacterial infection. β-glucan increased the levels of tight junction proteins occludin and zonula occludens-1 (ZO-1) in the colon (both *p* < 0.05, Fig. 4f). Consequently, β-glucan consumption attenuated serum LPS levels, which were elevated by HFFD (Fig. 4g), suggesting β-glucan enhancement of gut barrier integrity attenuated gut permeability to endotoxins. Next, we found that β-glucan consumption prevented the activation of TNF-α, IL-6, and IL-1β mRNA expression induced by the HFFD in the colon tissue (*p* < 0.05, Fig. 4h). As shortening colon length is associated with inflammation, we further found that β-glucan ameliorated colon length shortening induced by the HFFD (*p* < 0.05, Fig. 4i). Consistent with improved barrier function against endotoxin translocation, serum levels of proinflammatory cytokines were reduced by β-glucan compared with HFFD (*p* < 0.05, Fig. 4j). These results indicate that β-glucan prevented the damage of intestinal barrier integrity and the introduction to the circulation of bacterial products such as LPS, which subsequently promote intestinal and systemic immune reactions and inflammation.

### Long-term β-glucan supplementation prevented gut microbiota alteration in HFFD-fed mice

**Fig. 5:**
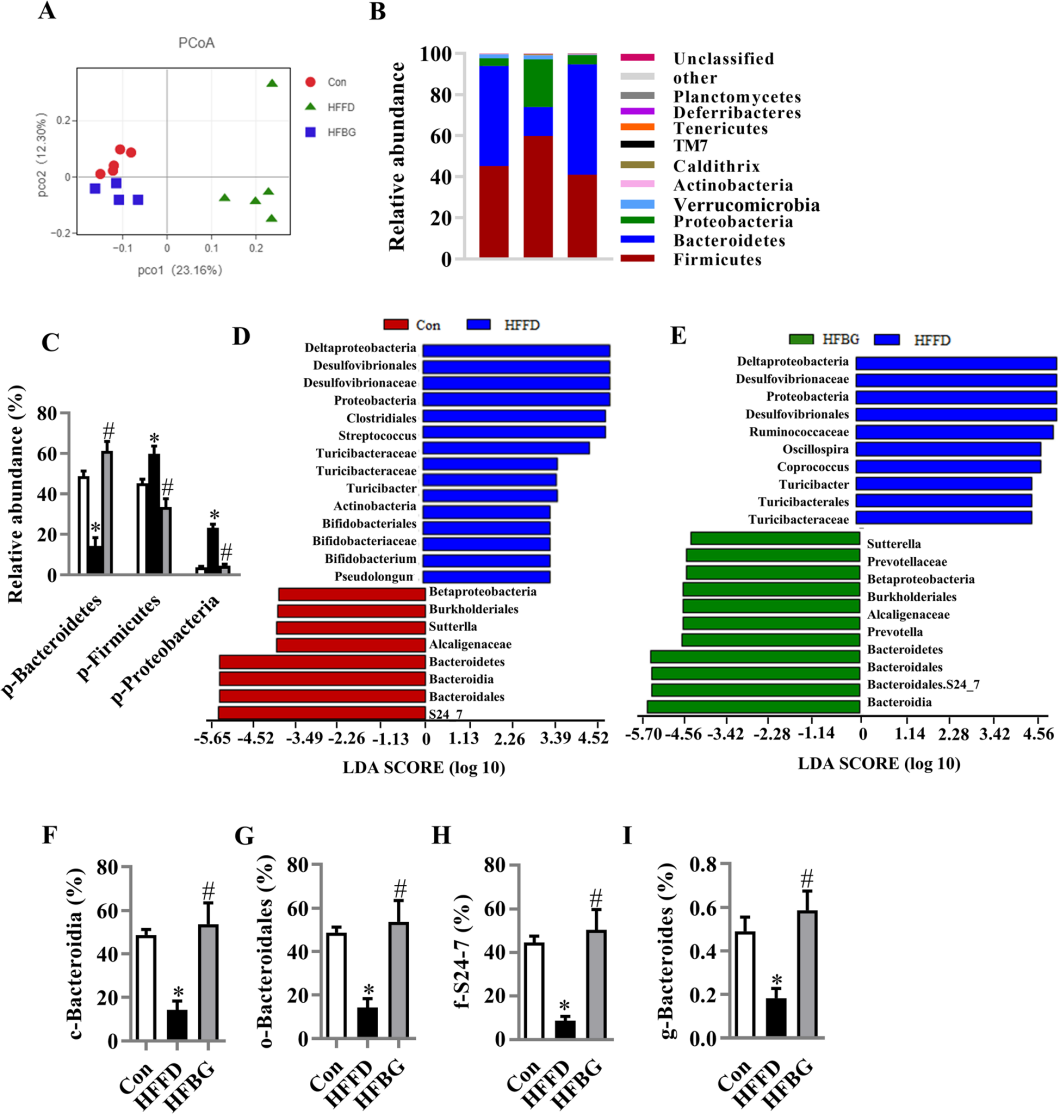
Long-term β-glucan supplementation prevented gut microbiota alteration in HFFD-induced obese mice. Cecal contents microbiota composition was analyzed by 16S rRNA gene sequencing (*n* = 4–5). **a** Principal coordinates analysis plot of unweighted UniFrac distances. **b** Composition of abundant bacterial phyla. **c** Comparison of the representative taxonomic abundance among Con, HFFD, and HFBG groups at phylum. **d** and **e** Linear discriminant analysis (LDA) effect size showing the most differentially significant abundant taxa enriched in microbiota from the Con vs. HFFD as well as HFBG vs. HFFD. **f**–**i** Comparison of the representative taxonomic abundance of Bacteroidetes among Con, HFFD, and HFBG groups at class (**g**), order (**h**), family (**i**), and genus (**j**). Values are mean ± SEM. ^*^p < 0.05 vs. Control (Con). ^#^*p* < 0.05 vs. high-fat and fiber-deficient (HFFD). Abbreviations: p, phylum; c, class; o, order; f, family; g, genus

To investigate the effects of HFFD, with or without β-glucan consumption, on gut microbiota, 16S rRNA gene sequencing was used to examine the effects of β-glucan on the diversity and composition of gut microbiota. HFFD significantly decreased α-diversity in the Shannon index (6.11 ± 0.13 vs. 5.36 ± 0.16, *p* = 0.0369). While β-glucan consumption (HFBG) and HFFD groups did not differ in the Shannon index (4.827 ± 0.26 vs. 5.36 ± 0.16, *p* = 0.171), principal component analysis (PCoA) of the UniFrac distance for β-diversity showed a clear separation between the HFFD groups with/without β-glucan consumption (Fig. 5a). The key phylotypes were distributed among three bacteria phyla, including Firmicutes, Bacteroidetes, and Proteobacteria (Fig. 5b and c). HFFD significantly decreased the relative abundance of Bacteroidetes and increased Proteobacteria compared with controls (both *p* < 0.05), while β-glucan restored these phyla to control group levels. Linear discriminant analysis effect size (LEfSe) indicated that bacteria belonging to the Bacteroidetes phylum, Bacteroidia class, Bacteroidales order, S24-7 family were differentially enriched in gut bacterial communities (LDA score > 3) between HFBG and HFFD groups (Fig. 5d and e). β-glucan supplementation fully prevented the HFFD-induced decrease in the relative abundances of these bacteria belonging to the Bacteroidetes phylum (Fig. 5f–i).

KEGG functional orthologs predicted by PICRUSt identified potential functional interactions between the gut microbiota and host among dietary groups in level one KEGG pathways, including cellular process, genetic information processing, environmental information processing, metabolism, human diseases, and organism system (e.g., environmental adaptation) (Table 1). In level two KEGG pathways, 8 functional orthologs were significantly altered in the HFFD group when compared with the control group. Importantly, β-glucan consumption was associated with marked microbial functional shifts in 12 functional orthologs, including cell growth and death, transport, and catabolism, translation in the cellular process; protein folding and associated genetic information processing; signal transduction in environmental information processing; amino acid, energy, cofactors and vitamins, and glycan biosynthesis, xenobiotics biodegradation in metabolism; neurodegenerative diseases; and environmental adaptation.

**Table 1:** Predicted KEGG functional pathway differences at level 2 inferred from 16S rRNA gene sequences using PICRUSt after chronic HFFD diet with or without β-glucan supplementation

KO functional categories						
Level_1	Level_2	Con mean% (SD%)	HFFD mean% (SD%)	HFBG mean% (SD%)	Con vs HFFD *p* value	HFBG vs HFFD *p* value
Cellular processes	Cell motility	3.491 (0.316)	4.891 (0.530)	3.084 (1.207)	0.008	_
Cellular processes	Cell growth and death	0.583 (0.013)	0.559 (0.026)	0.596 (0.055)	_	0.019
Cellular processes	Transport and catabolism	0.585 (0.042)	0.419 (0.028)	0.626 (0.065)	0.008	0.016
Genetic information processing	Translation	6.214 (0.205)	5.767 (0.271)	6.503 (0.645)	_	0.032
Genetic information processing	Transcription	3.149 (0.085)	3.526 (0.108)	2.943 (0.388)	0.015	_
Genetic information processing	Folding, sorting, and degradation	2.675 (0.074)	2.475 (0.157)	2.778 (0.333)	_	0.016
Environmental information processing	Signal transduction	2.123 (0.083)	2.694 (0.130)	2.000 (0.328)	0.012	0.032
Metabolism	Amino acid metabolism	11.09 (0.226)	10.490 (0.370)	11.311 (0.750)	_	0.032
Metabolism	Energy metabolism	6.565 (0.169)	6.134 (0.325)	6.812 (0.684)	_	0.016
Metabolism	Metabolism of cofactors and vitamins	4.793 (0.117)	4.618 (0.177)	4.915 (0.404)	_	0.016
Metabolism	Glycan biosynthesis and metabolism	3.402 (0.204)	2.666 (0.137)	3.592 (0.430)	0.008	0.016
Metabolism	Xenobiotics biodegradation and metabolism	1.787 (0.051)	1.929 (0.028)	1.724 (0.087)	0.016	0.001
Human diseases	Neurodegenerative diseases	0.175 (0.007)	0.181 (0.008)	0.172 (0.004)	0.027	0.032
Organismal systems	Environmental adaptation	0.186 (0.011)	0.218 (0.005)	0.177 (0.015)	0.016	0.032
Cellular processes	Cell motility	3.491 (0.316)	4.891 (0.530)	3.084 (1.207)	0.008	_
Cellular processes	Cell growth and death	0.583 (0.013)	0.559 (0.026)	0.596 (0.055)	_	0.019

Note: Data are given as mean% (SD%). *KEGG* Kyoto Encyclopedia of Genes and Genomes, *PICRUSt* Phylogenetic Investigation of Communities by Reconstruction of Unobserved States, *KO* KEGG Ortholog, *SD* standard deviation

### Short-term β-glucan supplementation prevented HFFD-induced gut microbiota alteration prior to the behavioral cognitive changes

**Fig. 6:**
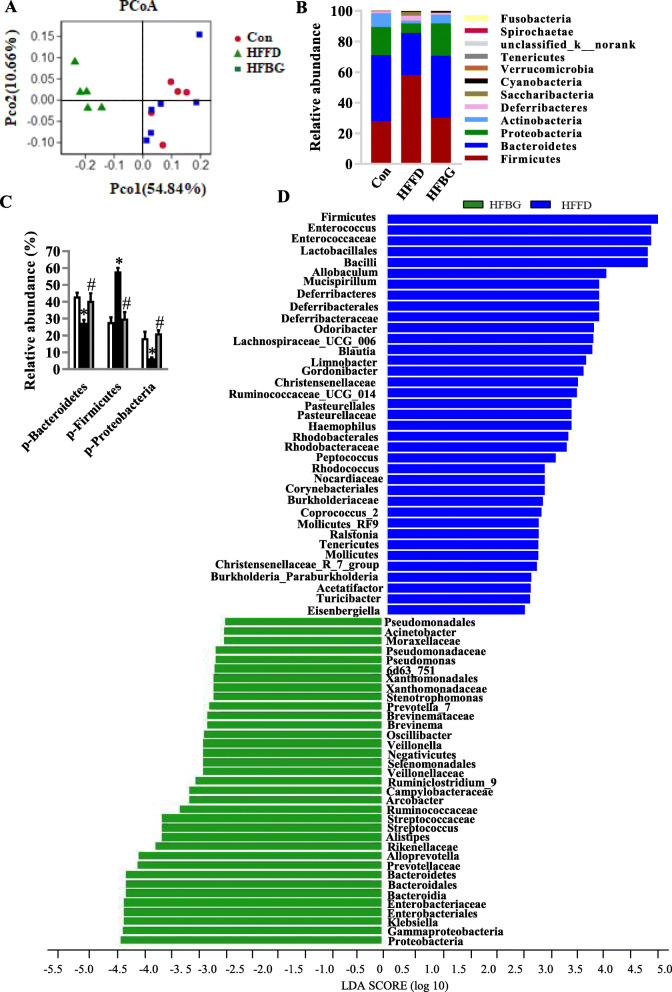
Short-term β-glucan supplementation prevented HFFD-induced gut microbiota alteration prior to cognitive improvement. Cecal content microbiota composition was analyzed by 16S rRNA gene sequencing (*n* = 5–6) (**a**–**d**). **a** Principal coordinates analysis plot of weighted UniFrac distances. **b** Composition of abundant bacterial phyla. **c** Comparison of the representative taxonomic abundance at phylum. **d** Linear discriminant analysis (LDA) effect size showing the most differentially significant abundant taxa enriched in microbiota from the HFBG and HFFD. Values are mean ± SEM. ^*^p < 0.05 vs. control (Con). ^#^*p* < 0.05 vs. high-fat and fiber-deficient diet (HFFD). Abbreviations: p, phylum

In order to investigate if β-glucan influences gut microbiota before the cognition changes, HFFD with and without β-glucan supplementation for 7 days were assessed. We found that short-term β-glucan supplementation did not change cognitive behavior in HFFD fed mice (Fig. S2A-D). Furthermore, short-term β-glucan supplementation suppressed over intake of energy induced by the HFFD (Fig. S2F) but did not alter body weight (Fig. S2E). The different effects of short-term β-glucan supplementation on body weight and food intake may be due to a time-limit, suggesting that the consequence of weight change occurs lately but is not consistent with instant energy intake alteration. However, by 16S rRNA gene sequencing analysis, the PCoA of the UniFrac distance revealed that HFFD feeding for 7 days dramatically changed gut microbial profile, with β-glucan-fed mice clustered apart from HFFD-fed mice sample (Fig. 6a), suggesting quick changes in the gut microbial profile are induced by short-term β-glucan consumption. In line with long-term consumption, short-term β-glucan consumption significantly increased Bacteroidetes phylum in mice on HFFD (Fig. 6b and c). β-glucan also prevented HFFD-increased Firmicutes and decreased Proteobacteria at phylum. LEfSe analysis showed that a higher abundance of phylum Bacteroidetes and Proteobacteria and their lower taxonomic level were notable in the β-glucan group (Fig. 6d), while phylum Firmicutes and its lower taxonomic levels were more significantly present in the HFFD group (Fig. S2G). The PICRUSt analysis showed that a total of 12 functional orthologs may interact between the gut microbiota and host metabolic regulation in the HFFD mice with short-term β-glucan supplementation (Table S2). Overall, short-term β-glucan supplementation did not change body weight and cognition; however, it changed the gut microbial profile. Therefore, short-term β-glucan supplementation prevented HFFD-induced gut microbiota alteration prior to the cognitive function and body weight changes.

### Microbiota ablation with antibiotics eliminated the effects of long-term β-glucan supplementation in abrogating endotoxemia and cognitive impairment

**Fig. 7:**
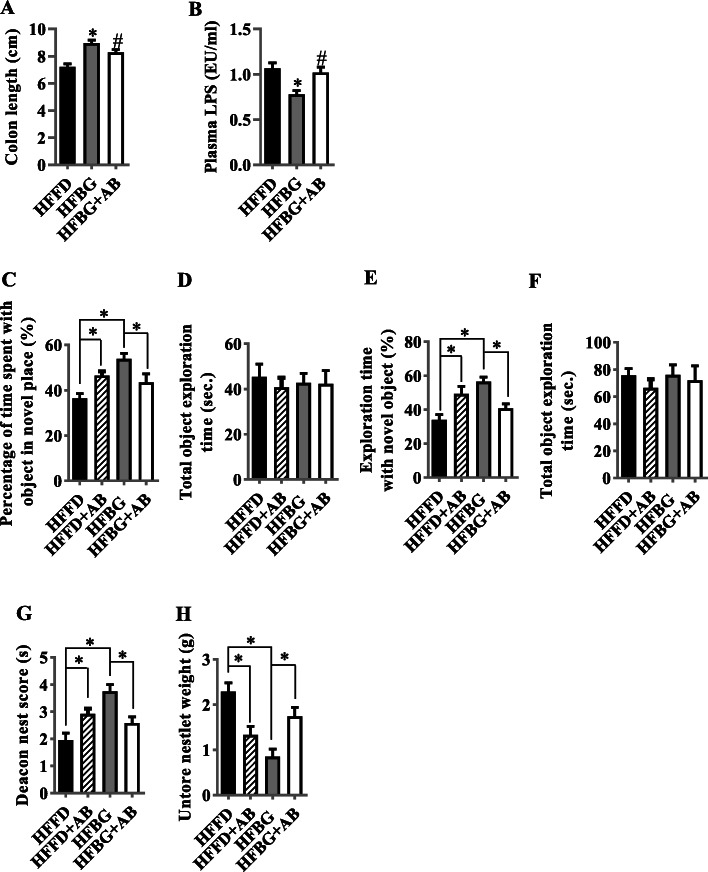
Microbiota ablation with antibiotics eliminated the effects of long-term β-glucan supplementation in improving endotoxemia and cognitive impairment. **a** The quantification of colon length was statistically analyzed (*n* = 10). **b** Serum LPS level (*n* = 10). ^*^p < 0.05 vs. high-fat and fiber-deficient diet (HFFD). ^#^*p* < 0.05 vs. β-glucan (HFBG). **c** Percentages of time spent with the object in the novel place. **d** Total object exploration time. **e** Percentage of time spent with the novel object. **f** Total object exploration time. **g** Nest score. **h** Untore nesting material (*n* = 12–15 per group). ^*^p < 0.05. Values are mean ± SEM

The above results suggest that the gut microbiota-brain axis plays an important role in β-glucan in improving cognition impairment induced by long-term HFFD feeding. To investigate the essential role of gut microbiota in β-glucan improving cognitive deficits, a cocktail of oral antibiotics was used to eliminate the β-glucan-induced gut microbiota effects. We found that the long-term β-glucan supplement group with a cocktail of oral antibiotics showed a 20-fold reduction in fecal bacterial load (Fig. S3). Antibiotics markedly reduced β-glucan promotion of colon length and endotoxemia (Fig. 7a and b). Furthermore, compared with the HFFD group, antibiotics significantly increased place recognition memory and novel object recognition memory in the object location and novel object recognition tests (Fig. 7c–f) and increased deacon nest score and decreased untore nestlet weight in the nest behavior test (Fig. 7g and h). While compared with the β-glucan supplement group, antibiotics significantly attenuated place and novel object recognition memory (Fig. 7c–f) and decreased deacon nest score and increased untore nestlet weight in the nest behavior test (Fig. 7g and h). These are all indicative of cognitive impairment and argue that the gut microbiota plays an essential role in mediating β-glucan’s positive impact on both the gut and, through that, on cognition.

## Discussion

**Fig. 8:**
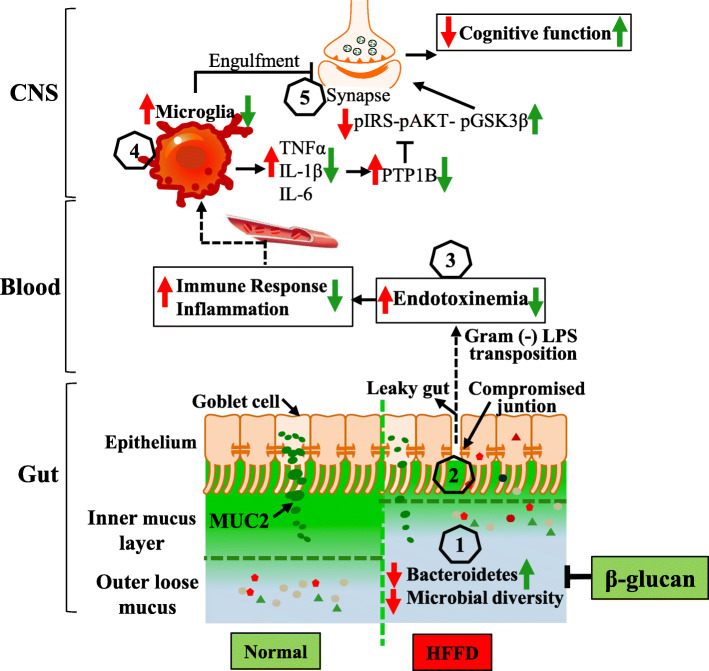
The interplay between the microbiota and the gut-brain axis in high-fat and fiber-deficient diet (HFFD) and β-glucan intervention. Gut microbiota contributes to regulating the gut-brain axis and maintaining health, while its alteration (decrease of Bacteroidetes and microbial diversity) due to HFFD is related to obesity and its adverse consequences on cognition (Steps 1–5). A β-glucan supplementation is thought to increase Bacteroidetes and gut microbiota (1), thereby, contribute to gut mucus and epithelial integrity and immune homeostasis (2); this attenuates the translocation of components of Gram-negative bacteria (3), which decreases the peripheral inflammatory tone and inhibits activation of microglia to neuroinflammation (4) and synapse engulfment in the CNS (5). Therefore, the supplement of β-glucan beneficially impacts on cognition, via restoration of gut microbiota and its regulatory role in the gut-brain axis

In the present study, we demonstrated a range of beneficial effects of β-glucan supplementation on the gut microbiota-brain axis by using a cognitive impairment mouse model induced by high-fat and fiber-deficient diet (Fig. 8). For the first time, we present evidence that long-term β-glucan supplementation ameliorated cognitive impairment assessed behaviorally and at the level of the hippocampus, and prevented major gut microbiota shift and mucosal barrier dysfunction assessed with a broad range of techniques. Furthermore, short-term β-glucan supplementation prevented microbial deviation from the normal state before significant cognitive improvement, suggesting the early response of gut microbiota to β-glucan intake. In addition, by the use of a broad-spectrum antibiotic intervention, the abrogation of β-glucan-induced improvement in cognitive function highlights the essential role of gut microbiota to mediate cognitive function and behavior.

The microbiota-gut-brain axis is considered to be a key regulator of neural function. For example, colonization with a conventional microbiota reverses the myelination alteration in germ-free mice both at the transcriptional and ultrastructural levels [30]. Further, obese-type microbiota transplantation disrupted intestinal barrier function and induced cognitive decline in mice [3]. In the present study, we found that long-term β-glucan consumption ameliorated a shift of gut microbiota composition induced by the HFFD. By using 16S rRNA gene sequencing analysis, after long-term β-glucan supplementation, the abundance of phylum Bacteroidetes was significantly increased, and the abundance of phylum Firmicutes and Proteobacteria were significantly decreased. The further LDA analysis showed that long-term β-glucan supplementation increased not only the Bacteroidetes at phylum, but also its lower taxa, such as Bacteroidia at class, Bacteroidales at order, S24-7 at family, and *Bacteroides* at genus in the HFFD-fed mice. In previous clinical studies, microbiota belonging to phylum Bacteroidetes has been associated with cognition and neurodegenerative diseases [31, 32], the corollary being that infants with high levels of gut *Bacteroides* at 1 year of age show higher cognitive ability at 2 years of age [31]. In a cross-sectional study, a lower abundance of *Bacteroides* at genus level is reported in the gut microbiota of dementia patients [32]. At the species level, *Bacteroides fragilis* was lower in patients with cognitive impairment [33]. Overall, these findings support that β-glucan supplementation improves gut microbiota composition, especially in Bacteroidetes, which may contribute to the prevention of cognition decline in diet-induced obese mice. In addition, the diversity of fecal microbiota is decreased in AD patients compared to cognitively healthy controls [34]. We found that gut microbiota diversity (Shannon index) was reduced in HFFD mice; however, β-glucan supplementation did not prevent these alterations in gut microbiota. Therefore, it is speculative that β-glucan improvement of gut microbiota composition, but not necessarily diversity per se, may be most critical for improved cognition.

In the present study, β-glucan supplementation for 7 days dramatically increased Bacteroidetes at phylum in HFFD mice. Similar to the effects during HFFD feeding, short-term β-glucan supplementation alone increased Bacteroidetes and decreased Firmicutes (Fig. S4, indicating that the abundance of members of Bacteroidetes is rapidly driven by the influx of polysaccharide β-glucan into the large intestine. Bacteroidetes comprise a dominant phylum in the human gut microbiota whose members thrive on dietary polysaccharides by polysaccharide utilization loci (PUL) [35, 36]. For example, PULs invariably encode a polysaccharide-binding protein at the outer membrane to capture polysaccharide [35]. Furthermore, PULs encode many polysaccharide lyases and hydrolases, which break down polysaccharide into oligosaccharide, and also encode transporters at the inner membrane of Bacteroidetes beneficial for the uptake of oligosaccharides [36]. Therefore, it is speculative that the host gut symbiont Bacteroidetes facilitate the acquisition, metabolism, and utilization of polysaccharide β-glucan and thereafter to promote the abundance of Bacteroidetes and its next taxa in gut microbiota. Overall, these findings suggest that β-glucan supplementation itself has the ability to prevent HFFD-induced gut microbiota shift (decreased proportion of Bacteroidetes), rather than body weight manipulation pushes the microbiota towards a new β-glucan-induced state.

Synergistically, Bacteroidetes have beneficial effects on their host intestinal mucosa and barrier function [37]. *Bacteroides thetaiotaomicron* increases colonic gene expression involved in the synthesis of mucosal glycans, such as α-1,2 fucosyltransferase, α-1,3-fucosyltransferase, glycosphingolipids, and O-glycans [38]. Oral administration of *Bacteroides fragilis* strengthen intestinal barrier and attenuate gut leakage in the autism mouse model [6]. In the present study, along with an increased abundance of Bacteroidetes, β-glucan prevented HFFD-induced degradation of the colonic mucosal barrier and microbiota encroachment. Thus, it is speculative that β-glucans may serve as platform elements, fermented by Bacteroidetes, to increase the production of mucosal glycans, thus enhancing the mucus layer overlying the intestinal epithelium to avoid epithelial damage. We further found that the intestinal tight junction proteins (occludin and ZO-1) were increased by β-glucan supplementation along with reduced systemic endotoxemia indicating increased integrity of the epithelial barrier and reduction of translocation of bacterial LPS into the circulation. Therefore, regular ingestion of β-glucan is integral to maintaining a healthy balance of microbes Bacteroidetes for improvement of intestinal integrity and reduction of intestinal and systemic inflammation in HFFD-fed mice. Pearson’s correlation analysis showed that the abundance of Bacteroidetes and its next levels of microbiota was significantly associated with colon tight junction proteins, serum LPS, hippocampal proinflammation, and cognitive behavior index (Fig. S5).

Over-exposure of LPS induces microglia activation and increases proinflammatory cytokines in the hippocampus of mice [7]. It is reported that endotoxin levels are increased threefold in the blood and two- or threefold in the brain of AD patients [39, 40]. In the present study, dietary β-glucan constrained microglia activation and reduced neuroinflammation in the hippocampus. Therefore, it is speculative that the enhancement of intestinal barrier function and reduction of LPS translocation might then be critical in β-glucan’s beneficial effects on the inflammatory cascade in the hippocampus. Proinflammatory cytokines, such as TNF-α, increase PTP1B transcription [8], block insulin signaling pIRS-pAKT-pGSK3β for synaptogenesis [9, 41, 42], and induce Tau phosphorylation, disrupting synapse formation and maintenance [29]. We found that long-term β-glucan supplementation downregulated PTP1B, improved insulin signaling pIRS-pAKT-pGSK3β, and inhibited Tau over-phosphorylation in the hippocampus. Thus β-glucan might exhibit its ability against HFFD-induced cognitive impairment by lowering neuroinflammation and restoration of insulin signaling and Tau neuronal proteins for synaptogenesis.

In our study, we found that the place discrimination index was decreased in HFFD feeding mice, which is consistent with other studies demonstrating that cognition was impaired in obese mice [43, 44]. For example, in Cope’s study, the place discrimination ratio was decreased in obese mice induced by a high-fat diet for 10 weeks compared with the control group, while both groups had similar total exploration time with the objects during the test phases [44]. Importantly, in our study, β-glucan supplementation increased place discrimination ratio in object location test compared with HFFD mice; however, there was no significant difference in the total exploration time with the objects during the test phases between two groups. Therefore, the higher place discrimination index in β-glucan supplementation group was not due to better general performance, but increased recognition memory. Furthermore, in this study, we found that mice on a HFFD built poorer quality nests. It is consistent with the previous study that high-fat diet-induced obese mice have shown decreased nest score [45]. We found that β-glucan improved nesting behavior in mice with HFFD feeding. The nesting behavior test can not only reflect cognition associated with brain regions in the hippocampus, but also may be influenced by several factors, such as thermoregulatory changes and the reduction in activity levels, due to obesity [45].

The dysregulation of synaptic ultrastructure has been implicated in cognitive impairment and AD [46, 47]. Using transmission electron microscopy, we observed synaptic structural plasticity and showed a reduction in PSD thickness and increased the width of the synaptic cleft in the HFFD group. Presynaptic, SYN, and postsynaptic, PSD95, are important for synaptic plasticity and synaptogenesis [48]. Reduction in SYN and PSD95 protein levels has been reported in the hippocampus of patients of AD or cognitive impairment [46, 47]. Here, dietary β-glucan supplementation prevented the reduction of SYN and PSD95 levels in the hippocampus of the mice with cognitive impairment. Therefore, it is speculative that β-glucan supplementation may contribute to the observed improvement in recognition memory and activities of daily living performance. It is reported that microglial activation participates in neurodegeneration, as exemplified by synaptic engulfment and pruning [49]. Here, we found that the HFFD induced a significantly higher volume of internalized PSD95 in microglia in the hippocampus than in the β-glucan supplementation groups again consistent with the β-glucan-induced improvement in synapse integrity and cognitive function.

Antibiotic cocktail treatment is a method that can be used to explore the effects of the microbiota on physiology and disease in mice [50]. In the previous study, the effects of antibiotics in control healthy group has been investigated reporting that antibiotics impair the cognition in control mice (C57 mice) with the disruption of the gut microbial community [51]. Here, we found that antibiotics improved cognitive impairment in HFFD obese mice. It suggests that antibiotics may attenuate HFFD-induced deleterious gut microbiota for cognition improvement. Our result is consistent with the previous study, in which antibiotics improved insulin resistance in the brain and multiple depressive-like and anxiety-like behaviors in high-fat diet-induced obese mice [52]. In contrast to the result of antibiotics in the HFFD group, we found that antibiotics abrogated β-glucan-induced improvement in cognitive function. These findings of antibiotic intervention suggest that gut microbiota regulated by β-glucan play an important role in cognition, rather than antibiotics directly affect the observed cognitive phenotypes.

β-glucans can be from various sources, which can have different chemical structures and vary in function. The β-glucans in cereal such as oat and barley are primarily in β(1,4) linkages separating shorter stretches of β(1,3) structure. While bacterial β-glucans consist of linear β(1,3) linkages, the β-glucans of mushrooms and fungi have a β(1,3) backbone branched with short β(1,6)-linked side chains. The cereal mixed-linkage β(1,3)/β(1,4)-glucan (MLG) is the specific site of hydrolysis by the MLG Utilization Locus (MLGUL), BoGH16MLG of Bacteroidetes lower taxa, such as *Bacteroides ovatus* [35]. Therefore, β(1,4)/β(1,3)-glucan is mainly fermented by gut microbiota in the lower gastrointestinal tract, potentially resulting in compositional and functional shifts in the gut microbiota [18, 53]. While only β(1,3) stimulates immunity and allows β(1,3) or β(1,3)/β(1,6)-glucans are recognized by dectin-1 and toll-like receptor-2 in various cells [54], it was reported that β-glucans from bacteria, mushroom, or fungi evoke the activation of immune cells and trigger the inflammatory response, such as activation of NF-κB [55]. In addition, low dose fungal β-glucan has been reported to promote highly immunoregulatory activity (FoxP3) and induce anti-inflammatory cytokines, including IL-10, TGFβ, and IL-2 via dectin-1 [56]. β(1,4)/β(1,3)-glucan is derived from oat or barley, which are common dietary fibers consumed in our daily life. In the present study, we investigated the effects of oral supplementation of β(1,4)/β(1,3)-glucan derived from oat on the gut microbiota and gut-brain axis to reduce neuroinflammation in the hippocampus of HFFD mice. However, it is unknown if oat β(1,4)/β(1,3)-glucans have direct effects on the immune cell, which requires further investigation.

Our previous studies have shown that oat β-glucans increase colon L cell-derived satiety hormone peptide Y-Y (PYY) in the clinical and rodent studies [57, 58]. It is reported that microbiota or its metabolites are associated with L cell activity and PYY secretion. For example, the abundance of *A. muciniphila* is associated with higher L cell number and activity [59]. Short-chain fatty acids, produced at high levels through the fermentation of fiber (such as β-glucan) by gut microbiota, potently stimulate PYY production in human enteroendocrine cells [60]. Previously, we found that oat β-glucans increase satiety by activation of the gut-hypothalamic (PYY-NPY) axis in diet-induced obesity in mice [61]. Therefore, these findings suggest that β-glucan as prebiotics can activate the gut-brain axis to increase satiety and reduce energy intake. Excessive dietary energy intake and insulin resistance have adverse effects on cognition reported in humans and rodent studies; however, energy restriction enhances neural plasticity by decreased inflammation and increased expression of synaptic plasticity-associated proteins [62]. Indeed, we found that the amount of energy intake on long-term β-glucan supplementation was negatively correlated with adverse metabolic parameters and positively correlated with cognitive parameters (Table S3). Therefore, the reduction of energy intake and improvement of metabolic parameters by β-glucan may contribute to cognition improvement. β-glucan supplementation not only regulates satiety via the gut-hypothalamic (PYY-NPY) axis. In the present study, we also found that oat β-glucan supplementation attenuated alterations caused by HFFD, including gut microbiota shift, gut barrier disruption, hippocampal inflammation, and abnormal synaptic ultrastructure. Therefore, β-glucan supplementation in maintaining the gut microbiota-brain (hippocampus) axis is important to prevent cognition decline induced by HFFD.

## Conclusions

The current results provide consistent evidence linking increased β-glucan intake to improved gut microbiota profile, intestinal barrier function, reduced endotoxemia, and enhanced cognitive function via more optimized synaptic and signaling pathways in critical brain areas. The use of an antibiotic intervention to abrogate the beneficial effects of β-glucan supplementation on gut microbiota also prevented the beneficial effect on cognitive impairment, suggesting the relationship between gut microbiota alteration and cognitive impairment may be causal. In addition to highlighting the adverse impact of Western diets on the gut-brain axis, the findings of this study suggest enhanced consumption of β-glucan-rich foods is an easily implementable nutritional strategy to attenuate the diet-induced cognitive decline.

## Methods

### Animals

C57BL/6 J male mice (11 weeks old) were obtained from the Experimental Animal Center of Xuzhou Medical University (SCXK_Su_2015-0009), and housed in environmentally controlled conditions (temperature 22 °C, 12 h light/dark cycle). After acclimatization to the laboratory conditions for 1 week, the mice were used for experiments in accordance with the Chinese Council on Animal Care Guidelines and approved by Institutional Animal Care Committee of Xuzhou Medical University.

### Long-term β-glucan supplementation experiment and cocktail antibiotic administration

The mice were randomly divided into 3 groups (*n* = 15 per group): (1) the control (Con) group were fed a grain-based rodent lab chow (LC, LabDiet 5010, 13.1% fat by energy, 15% neutral detergent fiber by weight), (2) the HFFD group were fed with a diet with high fat (55% by energy) and fiber deficient (50 g/kg from cellulose, low accessibility by gut microbiota, 5% fiber by weight); (3) the β-glucan (HFBG) group were fed with oat β-glucan derived from OatWell^TM^ oat bran (CreaNutrition, Switzerland) added into the HFFD (β-glucan 7% by weight, fiber content 14% by weight, detailed in Table S1). The dosage was according to our previous study [57] in which 7% oat β-glucan improved the regulation of the gut-hypothalamic (PYY_3-36_-Y2-NPY) axis. In addition, a fourth group (HFBG+AB, *n* = 12) was run in parallel with antibiotics (ampicillin 1 g/L, vancomycin 0.25 g/L, neomycin 1 g/L, and metronidazole 1 g/L) added to their drinking water with water renewed every 3 days for 15 weeks [63]. Mice fed a HFFD showed increased body weight from week 4 onwards, increased body fat accumulation and liver weight, and glucose intolerance (Fig. S1A-E). β-glucan supplementation attenuated these metabolic disturbances, but the final body weight and epididymal fat mass of the β-glucan supplement group were still higher than that of the control group. The lower body weight and improvement of other metabolic parameters may be due to the decreased energy intake by β-glucan supplements. The positive correlation was found between energy intake and body weight and other metabolic parameters (Supplement Table 3). Three cognitive behavior tests were performed after 15 weeks of intervention (described below). Three days following the last test, nine mice per group were sacrificed using CO_2_ asphyxiation. Blood, cecum content, colon, liver, fat (epididymal, inguinal, and interscapular masses), and brain tissues were immediately collected for the investigations of mRNA (left hippocampus) and protein expression (right hippocampus). The remaining mice (*n* = 6 per group) were sacrificed by CO_2_ asphyxiation and then transcardially perfused with PBS and paraformaldehyde for the studies of hippocampal immunohistochemistry and electron microscopy.

### Short-term β-glucan supplementation experiment

Similar to the long-term β-glucan supplementation experiment, the mice were randomly divided into three groups (*n* = 10 per group): the Con group, HFFD group, and HFBG group were respectively fed with the LC diet, HFFD, and 7% oat β-glucan in the HFFD for 7 days. After performing two cognitive behavior tests, the mice were sacrificed with a collection of cecum content for 16S rRNA gene sequencing of gut microbiota.

The long-term and short term β-glucan supplementation was repeated with 2 independent cohorts of mice.

### Behavioral tests

The object location, novel object recognition, and nesting behavior tests were performed in order to examine dietary effects on recognition memory and spontaneous rodent behaviors. Tests were conducted similar to previous studies [22, 24]. In the object location test, the place discrimination index (PDI) was calculated by using the formula: the time spent with the object moved to a novel place/the total time spent in exploring both the object moved to a novel place and the object remaining in the familiar place × 100. In the novel object recognition test, the novel object discrimination index (NODI) was calculated by using the formula: the time spent with the novel object/the total object exploration time × 100. For the nesting behavior test, the Deacon nest score and the untore nestlet weight were used to evaluate spontaneous rodent behavior (the ability of daily living).

### Microbial DNA extraction, PCR amplification, and Miseq sequencing in cecal contents

Genomic DNA amplification and sequencing were conducted as in our previous study [2]. Briefly, microbial DNA was extracted from the cecal contents of mice using the E.Z.N.A. stool DNA Kit (Omega Bio-tek, Norcross, GA, U.S.) according to the manufacturer’s protocols. The 16S rDNA V3-V4 region of the eukaryotic ribosomal RNA gene was amplified by PCR (95 °C for 2 min, followed by 27 cycles at 98 °C for 10 s, 62 °C for 30 s, and 68 °C for 30 s; and a final extension at 68 °C for 10 min) using primers 341F: CCTACGGGNGGCWGCAG; 806R: GGACTACHVGGGTATCTAAT, where the barcode is an eight-base sequence unique to each sample. PCR reactions were performed in triplicate 50 μL mixture containing 5 μL of 10 × KOD Buffer, 5 μL of 2.5 mM dNTPs, 1.5 μL of each primer (5 μM), 1 μL of KOD polymerase, and 100 ng of template DNA Amplicons extracted from 2% agarose gels and purified using the AxyPrep DNA Gel Extraction Kit (Axygen Biosciences, Union City, CA, USA) according to the manufacturer’s instructions and quantified using QuantiFluor-ST (Promega, USA). Purified amplicons were pooled in equivalent molar and paired-end sequences (2 × 250) on an Illumina platform according to the standard protocols.

### Measurement of serum cytokines

ELISA kits were used to measure TNF-α, IL-6, and IL-1β of serum according to the manufacturer’s instructions (Thermo Fisher, USA).

### Lipopolysaccharide determination

The concentration of circulating serum LPS was measured by enzyme-linked immunosorbent assay (Limulus assay kit, Cat.18110115, China). The absorbance was measured at 545 nm using a spectrophotometer, with measurable concentrations ranging from 0.1 to 1.0 EU/ml. All samples for LPS measurements were performed in duplicate.

### Intraperitoneal glucose tolerance test

The intraperitoneal glucose tolerance test (IPGTT) was conducted as we have previously described [64]. Briefly, mice were fasted overnight followed by an intraperitoneal injection of glucose (2 g/kg). Blood samples were obtained from the tail vein at 0, 30, 60, 90, and 120 min following the injection of glucose. Blood glucose levels were measured with a glucose meter (Accu-Chek).

### Thickness measurements of the colonic mucus layer

Post Carnoy’s fixation, the methanol-stored colon samples were embedded in paraffin, cut into thin sections (5 μm), and deposited on glass slides. Alcian blue staining was performed by the protocols as previously published [65]. The thickness of the colonic sections was then measured (10 measurements per section/2 sections per animal/5 animals per group) using ImageJ after cross-validation using anti-MUC2 staining.

### Bacteria localization by FISH staining

The staining of bacteria localization at the surface of the intestinal mucosa was conducted as previously described [66]. Briefly, transverse colonic tissues full of fecal material were placed in methanol-Carnoy’s fixative solution (60% methanol, 30% chloroform, 10% glacial acetic acid) for a minimum of 3 h at room temperature. Tissues were then washed in methanol 2× 30 min, ethanol 2× 20 min, and xylene 2× 20 min, and embedded in paraffin for 5-μm sections on glass slides. The tissue sections were dewaxed by preheating at 60 °C for 10 min, followed by xylene 60 °C for 10 min, xylene for 10 min, and 100% ethanol for 10 min. Deparaffinized sections were incubated at 37 °C overnight with EUB338 probe (5′-GCTGCCTCCCGTAGGAGT-3′) diluted to 10 μg/mL in hybridization buffer (20 mM Tris–HCl, pH 7.4, 0.9 M NaCl, 0.1% SDS, 20% formamide). After incubating with wash buffer (20 mM Tris–HCl, pH 7.4, 0.9 M NaCl) for 10 min and 3× 10 min in PBS sequentially, the slides were mounted in DAPI-containing mounting medium.

### Immunohistochemistry

MUC2 in the colon was detected by staining the colonic tissue sections (5 μm) with anti-MUC2 antibody (Abclonal, A14659) diluted 1:500 in TBS and goat-anti-rabbit Alexa 488 conjugated antibody (1:1000) (Invitrogen, A32731) in TBS. At a temperature of − 18 °C, 20 μm frozen brain sections (hippocampus) were cut using a cryostat from Bregma − 3.3 to − 4.16 mm according to a standard mouse brain atlas [67]. The brain slices were blocked with 10% goat normal serum for 15 min at room temperature and then incubated with the primary antibodies at 4 °C overnight. The primary antibody anti-Iba1 (Wako, 019–19741) and PSD95 (CST, 3450) were used. After washing with PBS, the sections were incubated with the secondary antibodies at 37 °C for 1 h. The secondary antibody Alexa Fluor® 594 (abcom 150160) and Alexa Fluor® 488 (ab150117) were used. Finally, the sections were counterstained with DAPI (Sigma, D9542). The morphology of microglia in the CA1, CA3, and DG of the hippocampus was then imaged with a microscope (OLYMPUS IX51). The hippocampal CA1 area in brain tissue sections was imaged by a Leica SP8 confocal microscope system equipped with a × 63 oil immersion objective (Leica, Germany) by using identical light intensity and exposure settings in stacks (z-step 0.1 μm). The images of contact between microglia and postsynaptic structures in identical × 60 image stacks from sections double-labeled for Iba1 and PSD95 were processed by the LAS X software (Leica, Germany).

### Quantitative RT-PCR

Total RNA was extracted from tissues homogenized in Trizol (Thermo Fisher Scientific, Waltham, MA, USA). One microgram of purified RNA was used for RT-PCR to generate cDNA with a High-Capacity cDNA Reverse Transcription Kit (Takara, Dalian, China), and the resulting cDNA was used for quantitative PCR on a real-time PCR detection system (Bio-Rad, Hercules, CA, USA). The relative mRNA expression level was determined with the 2-ΔΔCt method with GAPDH as the internal reference control. Primer sequences were as follows: mTNFα--forward (F): CTTGTTGCCTCCTCTTTTGCTTA, mTNFα--reverse (R): CTTTATTTCTCTCAATGACCCGTAG; mIL-1β--forward (F): TGGGAAACAACAGTGGTCAGG, mIL-1β--reverse (R): CTGCTCATTCACGAAAAGGGA; mIL-6--forward (F): TCACAGAAGGAGTGGCTAAGGACC, mIL-6--reverse (R): ACGCACTAGGTTTGCCGAGTAGAT; mGAPDH--forward (F): AGAAGGTGGTGAAGCAGGCATC, mGAPDH--reverse (R): CGAAGGTGGAAGAGTGGGAGTTG; mReg3γ--forward (F): 5′TTCCTGTCCTCCATGATCAAA-3′, mReg3γ--reverse (R): 5′CATCCACCTCTGTTGGGTTC-3.

### Western blotting

Mouse colon and hippocampus were homogenized in ice-cold RIPA lysis buffer, supplemented with complete EDTA-free protease inhibitor cocktail and PhosSTOP Phosphatase Inhibitor. The homogenate was sonicated six times for 4 s, at 6 s intervals on ice and then centrifuged at 12,000 *g* for 20 min at 4 °C. The supernatant was collected, and the protein concentration was quantitated by BCA assay. Equal amounts of protein were separated by sodium dodecyl sulfate-polyacrylamide gel electrophoresis (SDS-PAGE) and transferred onto polyvinylidene difluoride (PVDF) membranes. The membrane was blocked with 5% non-fat milk at room temperature for 1 h, and then incubated with the primary antibody at 4 °C overnight. These primary antibodies were included: anti-occludin (Abcam, ab167161), anti-ZO1 (Abcam, ab96587), anti-p-IRS-1 (Ser307) (CST, 2381T), anti-IRS-1 (CST, 2382S), anti-Iba1 (Wako, 019–19741), anti-p-GSK-3β(Ser9) (CST, 9322S), anti-GSK-3-β (CST, 12456T), anti-p-AKT(Ser473) (CST, 4060T), anti-AKT (CST, 4691T), anti-Tau5 (Abcam, ab80579), anti-p-Tau (S202 + T205) (Abcam, ab80579), anti-synaptophysin (Abcam, ab32127), anti-PSD95 (CST, 3450), anti-PTP1B (Abcam, ab189179), GAPDH (ABclonal, AC033), and β-actin (ABclonal, AC026). Following 3 washes in TBST, the membrane was incubated with HRP-inked anti-rabbit IgG secondary antibody (CST, 7074) or HRP-linked anti-mouse IgG secondary antibody (CST, 7076S) at room temperature for 1 h. After washing 3 times with TBST, the protein bands were detected with Clarity™ ECL western blot substrate (Bio-Rad, 1,705,060) and visualized using the ChemiDoc Touch imaging system (Bio-Rad).

### Transmission electron microscopy

The left side of the hippocampal CA1 was taken and rapidly fixed in glutaraldehyde. After fixation for 24 h, the hippocampal tissues of control, HFFD, and HFBG mice were quickly dissected and separated into thin slices. They were fixed immediately with 2.5% glutaraldehyde at 4 °C overnight. Washed 3 times in phosphate-buffered saline (PBS), these slices were fixed in 1% osmium tetroxide, stained with 2% aqueous solution of uranyl acetate, and then dehydrated with different concentrations of ethanol and acetone gradient. Finally, they were embedded in epoxy resin. Ultra-thin sections (70 nm) were cut with ultramicrotome, collected on copper grids, and then stained with 4% uranyl acetate and lead citrate. Synapses are classified into asymmetric and symmetric synapses, or Gray I type and Gray II type synapses, which are considered to mediate excitatory and inhibitory transmission, respectively. Asymmetric synapses have prominent postsynaptic densities and relatively wide synaptic clefts while symmetric synapses are with pre- and postsynaptic densities of equal thickness and narrower synaptic clefts. In the present study, asymmetric synapses were examined for excitatory synaptic measurement. The PSD thickness was evaluated as the length of a perpendicular line traced from the postsynaptic membrane to the most convex part of the synaptic complex. The widths of the synaptic clefts (SCs) were estimated by measuring the widest and narrowest portions of the synapse and then averaging these values.

### Statistical analysis

Data were analyzed using the statistical package SPSS (Version 20, Chicago, USA). After data were tested for normality, the differences among the intervention groups were determined using a one way analysis of variance (ANOVA) followed by the post hoc Tukey-Kramer test. A *p* value of < 0.05 was considered to be statistically significant. For 16S rRNA gene sequencing analysis, all reads were deposited and grouped into operational taxonomic units (OTU) at a sequence identity of 97%, and the taxonomic affiliation of the OTUs was determined with quantitative insights into microbial ecology (QIIME, version 1.8.0) against the Greengenes database version 13.8. Based on the Kyoto Encyclopedia of Genes and Genomes (KEGG) functional pathway, the predicted functional composition of the intestinal microbiome was inferred for each sample using Phylogenetic Investigation of Communities by Reconstruction of Unobserved States (PICRUSt) [68]. Statistical analyses were conducted with STAMP, and functional differences in orthologs among groups were assessed by a one way ANOVA followed by post hoc Tukey-Kramer multiple comparisons.

## Supplementary information

**Additional file 1: Figure S1.** High-fat and fibre-deficient (HFFD) diet induced metabolic syndrome in mice, which were to some degree attenuated by long-term β-glucan supplementation. (A) Body weight over time (n=15). (B) Average energy intake (n=15). (C) Fat pad weight (n=9). (D) liver mass (n=9) and representative images of livers. (E) Glucose tolerance test and area under curve (AUC) calculated (n=10). Values are mean ± SEM. *p < 0.05 vs. Con. #p < 0.05 vs. HFFD. $p <0.05 vs. Con. eWAT: epididymal white adipose tissue; iWAT: inguinal white adipose tissue; iBAT: interscapular brown adipose tissue. Figure S2. Short-term β-glucan (HFBG) supplementation for 7 days did not affect body weight and cognitive behaviours in mice. (A) Percentages of time spent with the object in the novel place to total object exploration time. (B) Total object exploration time. (C) Nest score. (D) Untore nestler weight (amount of untore nesting material). (E) Body weight. (F) Average energy intake. (G) Linear discriminant analysis (LDA) effect size showing the most differentially significant abundant taxa enriched in microbiota from the control (Con) vs. HFFD. *p < 0.05 vs. Control (Con). ^#^*P* < 0.05 vs. high-fat and fibre-deficient (HFFD). Figure S3. Antibiotics significantly decreased bacterial DNA of faces in HFBG mice (n = 6). *p < 0.05 β-glucan supplementation in HFFD (HFBG) group vs. β-glucan supplement with antibiotics (HFBG+AB) group. Figure S4. Short-term β-glucan supplementation (BG) alone affected the gut microbiota. Caecal contents microbiota composition was analyzed by 16S rRNA gene sequencing (n=5-6). β-glucan supplementation increased Bacteroidetes and decreased Firmicutes (A). Principal coordinates analysis plot of weighted UniFrac distances (B). *p < 0.05 vs. Control (Con). Figure S5. Pearson’s correlations between Bacteroidetes and its down taxa, and the parameters of gut, brain and cognitive behaviour. *p < 0.05, **p < 0.01, ***p < 0.001, ****p < 0.0001.

**Additional file 2: Table S1.** Composition of the diets including β-glucan derived from oat bran ingredient

**Additional file 3: Table S2.** Predicted KEGG functional pathway differences at level 2 inferred from 16S rRNA gene sequences using PICRUSt after acute HFFD diet with or without β-glucan supplementation.

**Additional file 4: Table S3.** Pearson correlations between energy intake and metabolic and behavior parameters.

## Data Availability

The sequencing data of the 16S rRNA gene in this study are available in the Sequence Read Archive (SRA) under project number PRJNA611892.
