# Supplementary material for: β-glucan attenuates cognitive impairment via the gut-brain axis in diet-induced obese mice
Source: Microbiome. 2020 Oct 2;8:143. doi: 10.1186/s40168-020-00920-y (PMC7532656; doi:10.1186/s40168-020-00920-y)
Supplement: Supplementary file 2 — Additional file 1: Figure S1. High-fat and fibre-deficient (HFFD) diet induced metabolic syndrome in mice, which were to some degree attenuated by long-term β-glucan supplementation. (A) Body weight over time (n=15). (B) Average energy intake (n=15). (C) Fat pad weight (n=9). (D) liver mass (n=9) and representative images of livers. (E) Glucose tolerance test and area under curve (AUC) calculated (n=10). Values are mean ± SEM. *p < 0.05 vs. Con. #p < 0.05 vs. HFFD. $p <0.05 vs. Con. eWAT: epididymal white adipose tissue; iWAT: inguinal white adipose tissue; iBAT: interscapular brown adipose tissue. Figure S2. Short-term β-glucan (HFBG) supplementation for 7 days did not affect body weight and cognitive behaviours in mice. (A) Percentages of time spent with the object in the novel place to total object exploration time. (B) Total object exploration time. (C) Nest score. (D) Untore nestler weight (amount of untore nesting material). (E) Body weight. (F) Average energy intake. (G) Linear discriminant analysis (LDA) effect size showing the most differentially significant abundant taxa enriched in microbiota from the control (Con) vs. HFFD. *p < 0.05 vs. Control (Con). #P < 0.05 vs. high-fat and fibre-deficient (HFFD). Figure S3. Antibiotics significantly decreased bacterial DNA of faces in HFBG mice (n = 6). *p < 0.05 β-glucan supplementation in HFFD (HFBG) group vs. β-glucan supplement with antibiotics (HFBG+AB) group. Figure S4. Short-term β-glucan supplementation (BG) alone affected the gut microbiota. Caecal contents microbiota composition was analyzed by 16S rRNA gene sequencing (n=5-6). β-glucan supplementation increased Bacteroidetes and decreased Firmicutes (A). Principal coordinates analysis plot of weighted UniFrac distances (B). *p < 0.05 vs. Control (Con). Figure S5. Pearson’s correlations between Bacteroidetes and its down taxa, and the parameters of gut, brain and cognitive behaviour. *p < 0.05, **p < 0.01, ***p < 0.001, ****p < 0.0001. [file 40168_2020_920_MOESM1_ESM.pptx]

## Slide 1
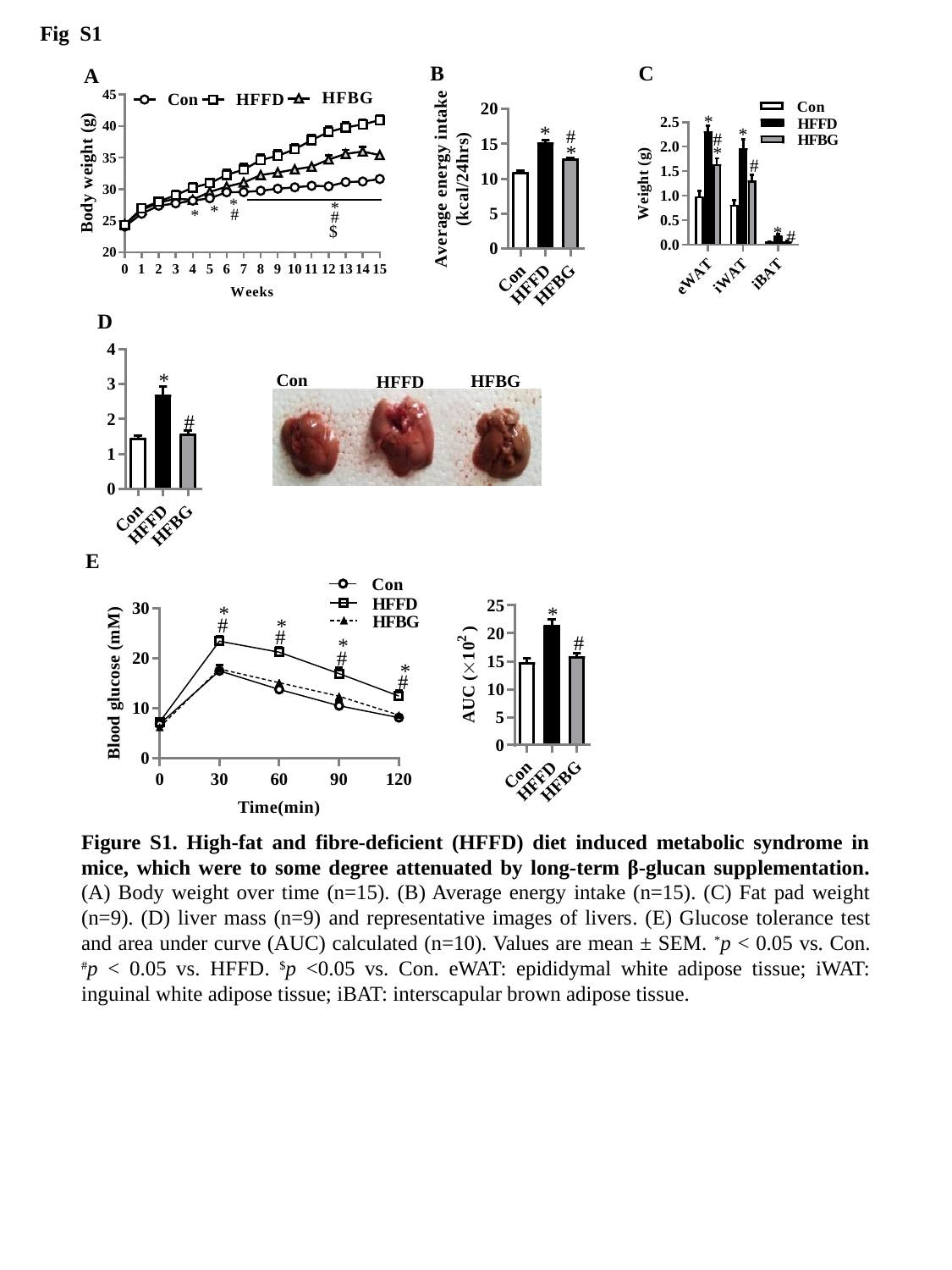

Fig S1
B
C
A
D
Con
HFBG
HFFD
E
Figure S1. High-fat and fibre-deficient (HFFD) diet induced metabolic syndrome in mice, which were to some degree attenuated by long-term β-glucan supplementation. (A) Body weight over time (n=15). (B) Average energy intake (n=15). (C) Fat pad weight (n=9). (D) liver mass (n=9) and representative images of livers. (E) Glucose tolerance test and area under curve (AUC) calculated (n=10). Values are mean ± SEM. *p < 0.05 vs. Con. #p < 0.05 vs. HFFD. $p <0.05 vs. Con. eWAT: epididymal white adipose tissue; iWAT: inguinal white adipose tissue; iBAT: interscapular brown adipose tissue.

## Slide 2
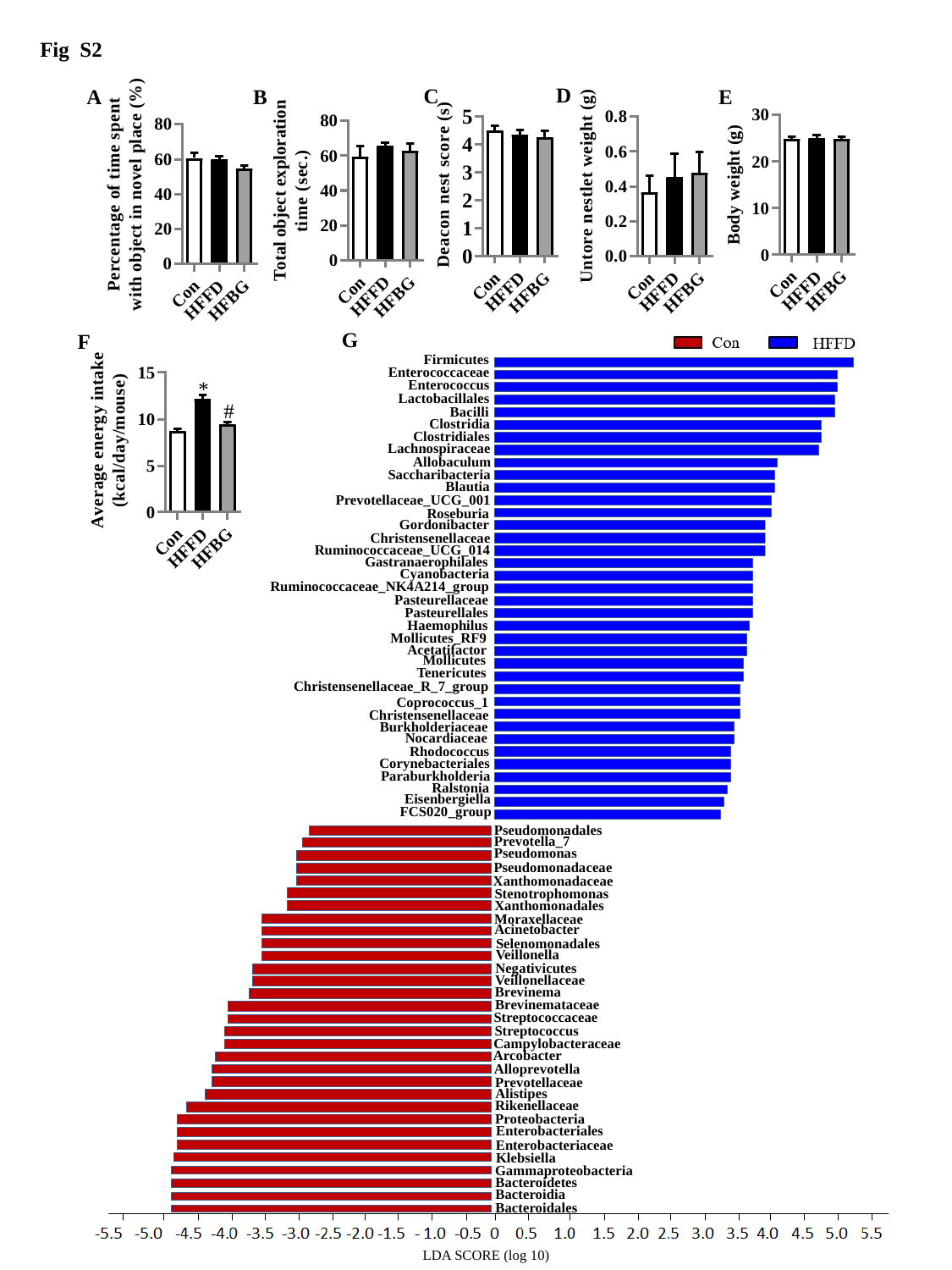

Fig S2
A
D
C
E
B
G
F
D
Firmicutes
Enterococcaceae
Enterococcus
Lactobacillales
Bacilli
Clostridia
Clostridiales
Lachnospiraceae
Allobaculum
Saccharibacteria
Blautia
Prevotellaceae_UCG_001
Roseburia
Gordonibacter
Christensenellaceae
Ruminococcaceae_UCG_014
Gastranaerophilales
Cyanobacteria
Ruminococcaceae_NK4A214_group
Pasteurellaceae
Pasteurellales
Haemophilus
Mollicutes_RF9
Acetatifactor
Mollicutes
Tenericutes
Christensenellaceae_R_7_group
Coprococcus_1
Christensenellaceae
Burkholderiaceae
Nocardiaceae
Rhodococcus
Corynebacteriales
Paraburkholderia
Ralstonia
Eisenbergiella
FCS020_group
Pseudomonadales
Prevotella_7
Pseudomonas
Pseudomonadaceae
Xanthomonadaceae
Stenotrophomonas
Xanthomonadales
Moraxellaceae
Acinetobacter
Selenomonadales
Veillonella
Negativicutes
Veillonellaceae
Brevinema
Brevinemataceae
Streptococcaceae
Streptococcus
Campylobacteraceae
Arcobacter
Alloprevotella
Prevotellaceae
Alistipes
Rikenellaceae
Proteobacteria
Enterobacteriales
Enterobacteriaceae
Klebsiella
Gammaproteobacteria
Bacteroidetes
Bacteroidia
Bacteroidales
LDA SCORE (log 10)

## Slide 3
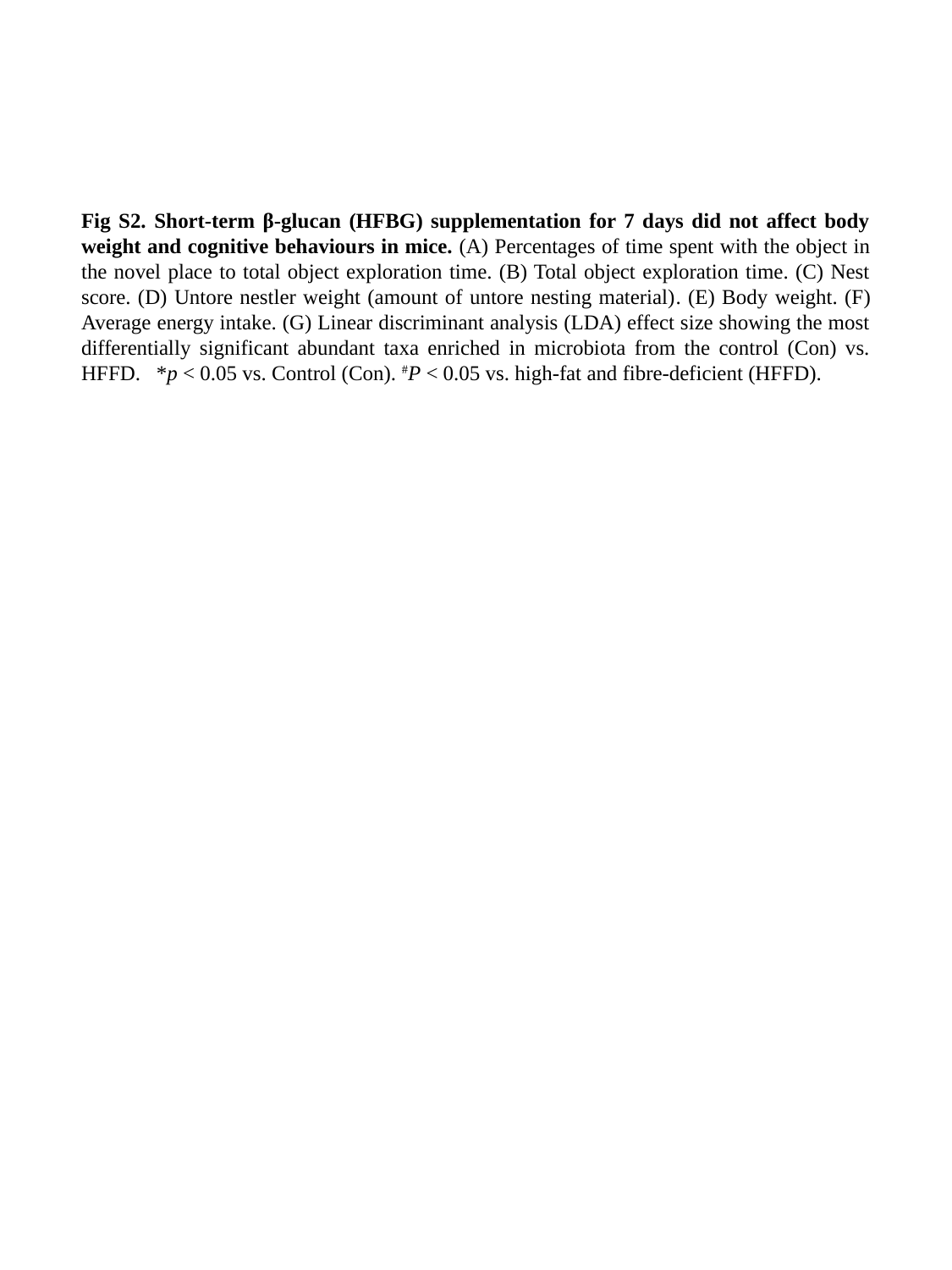

Fig S2. Short-term β-glucan (HFBG) supplementation for 7 days did not affect body weight and cognitive behaviours in mice. (A) Percentages of time spent with the object in the novel place to total object exploration time. (B) Total object exploration time. (C) Nest score. (D) Untore nestler weight (amount of untore nesting material). (E) Body weight. (F) Average energy intake. (G) Linear discriminant analysis (LDA) effect size showing the most differentially significant abundant taxa enriched in microbiota from the control (Con) vs. HFFD. *p < 0.05 vs. Control (Con). #P < 0.05 vs. high-fat and fibre-deficient (HFFD).

## Slide 4
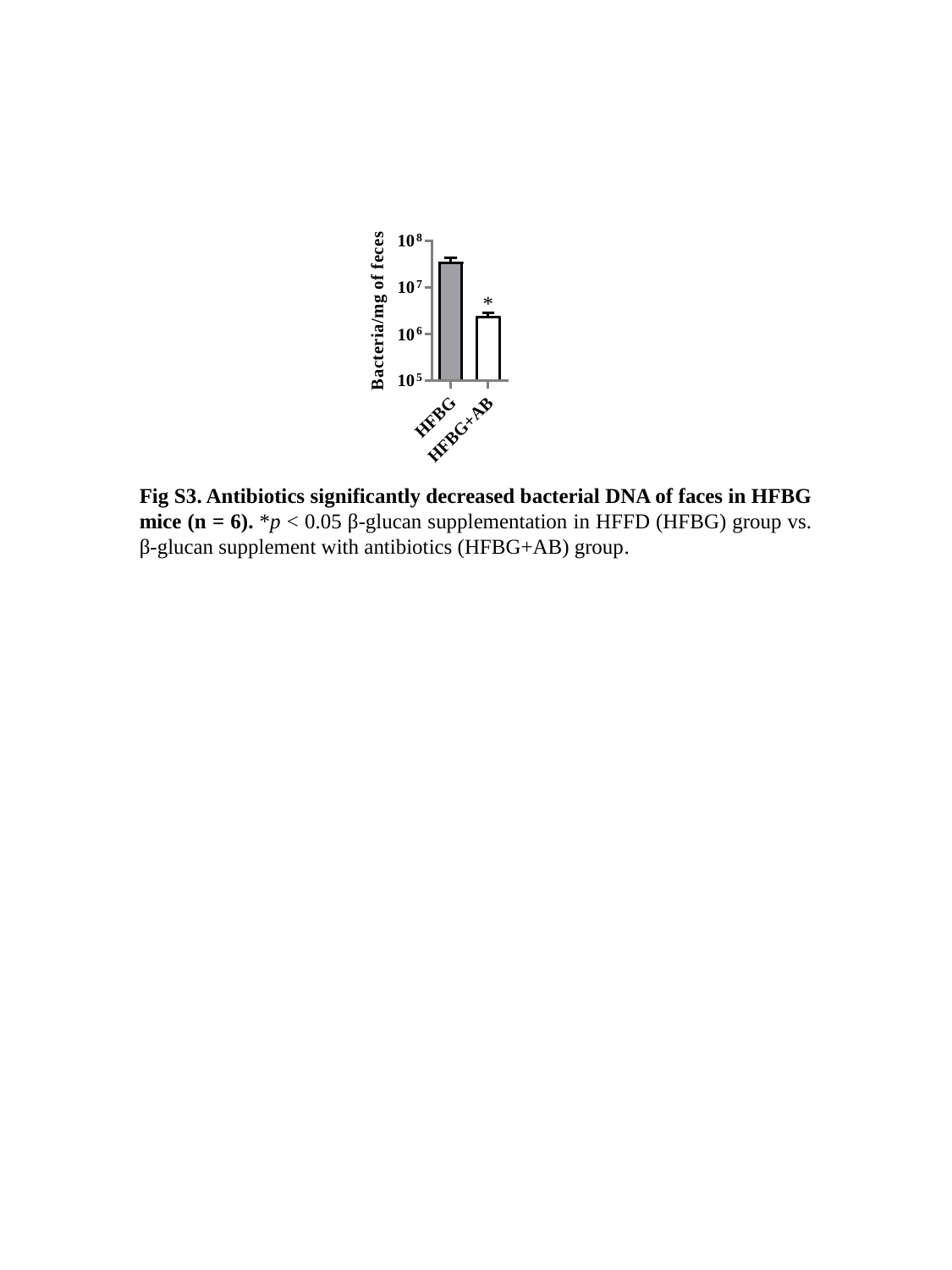

Fig S3. Antibiotics significantly decreased bacterial DNA of faces in HFBG mice (n = 6). *p < 0.05 β-glucan supplementation in HFFD (HFBG) group vs. β-glucan supplement with antibiotics (HFBG+AB) group.

## Slide 5
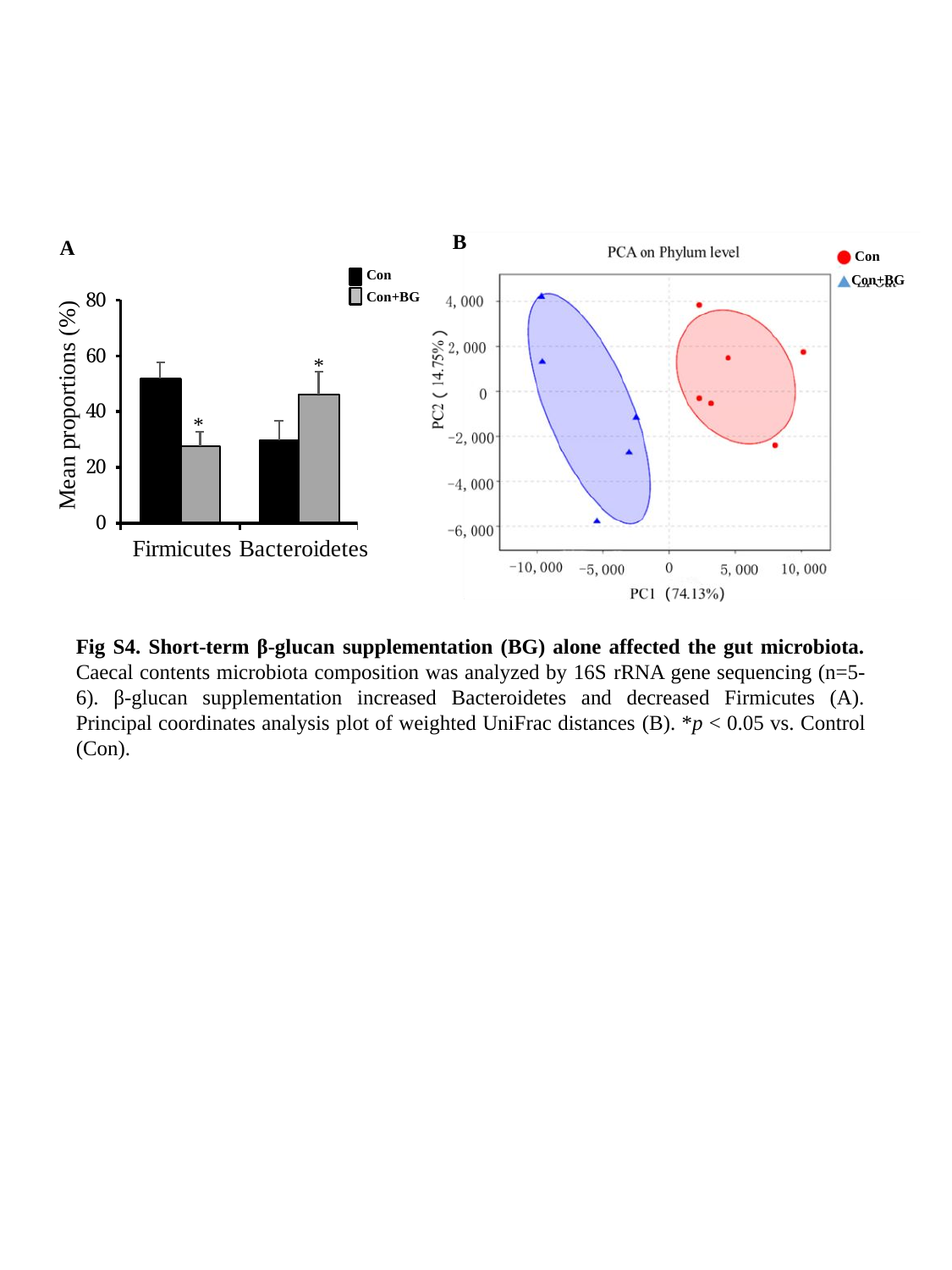

B
A
Con
Con
Con+BG
Con+BG
*
*
Fig S4. Short-term β-glucan supplementation (BG) alone affected the gut microbiota. Caecal contents microbiota composition was analyzed by 16S rRNA gene sequencing (n=5-6). β-glucan supplementation increased Bacteroidetes and decreased Firmicutes (A). Principal coordinates analysis plot of weighted UniFrac distances (B). *p < 0.05 vs. Control (Con).

## Slide 6
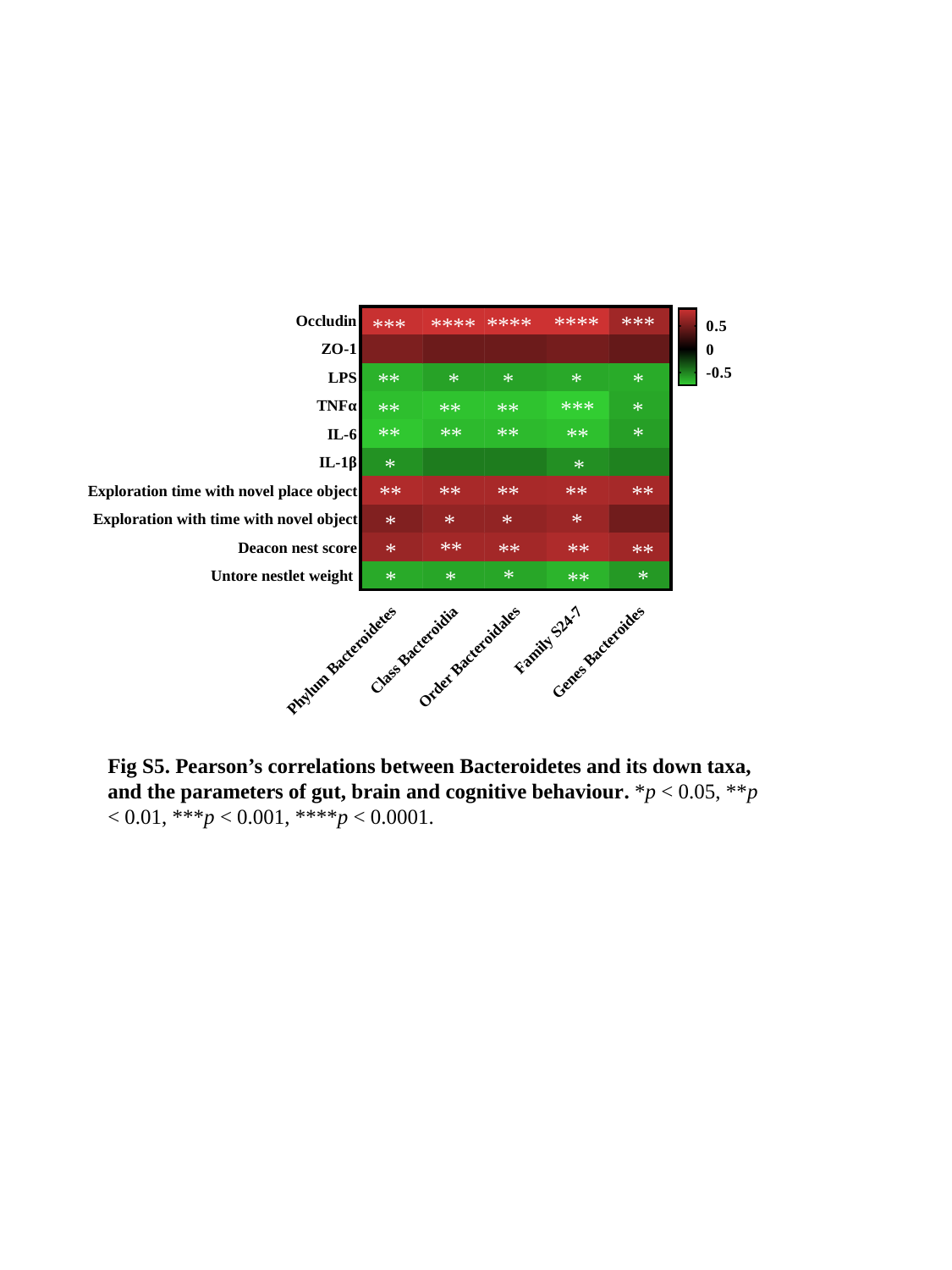

Fig S5. Pearson’s correlations between Bacteroidetes and its down taxa, and the parameters of gut, brain and cognitive behaviour. *p < 0.05, **p < 0.01, ***p < 0.001, ****p < 0.0001.
