# Supplementary material for: β-glucan attenuates cognitive impairment via the gut-brain axis in diet-induced obese mice
Source: Microbiome. 2020 Oct 2;8:143. doi: 10.1186/s40168-020-00920-y (PMC7532656; doi:10.1186/s40168-020-00920-y)
Supplement: Supplementary file 3 — Additional file 2: Table S1. Composition of the diets including β-glucan derived from oat bran ingredient [file 40168_2020_920_MOESM2_ESM.pptx]

## Slide 1
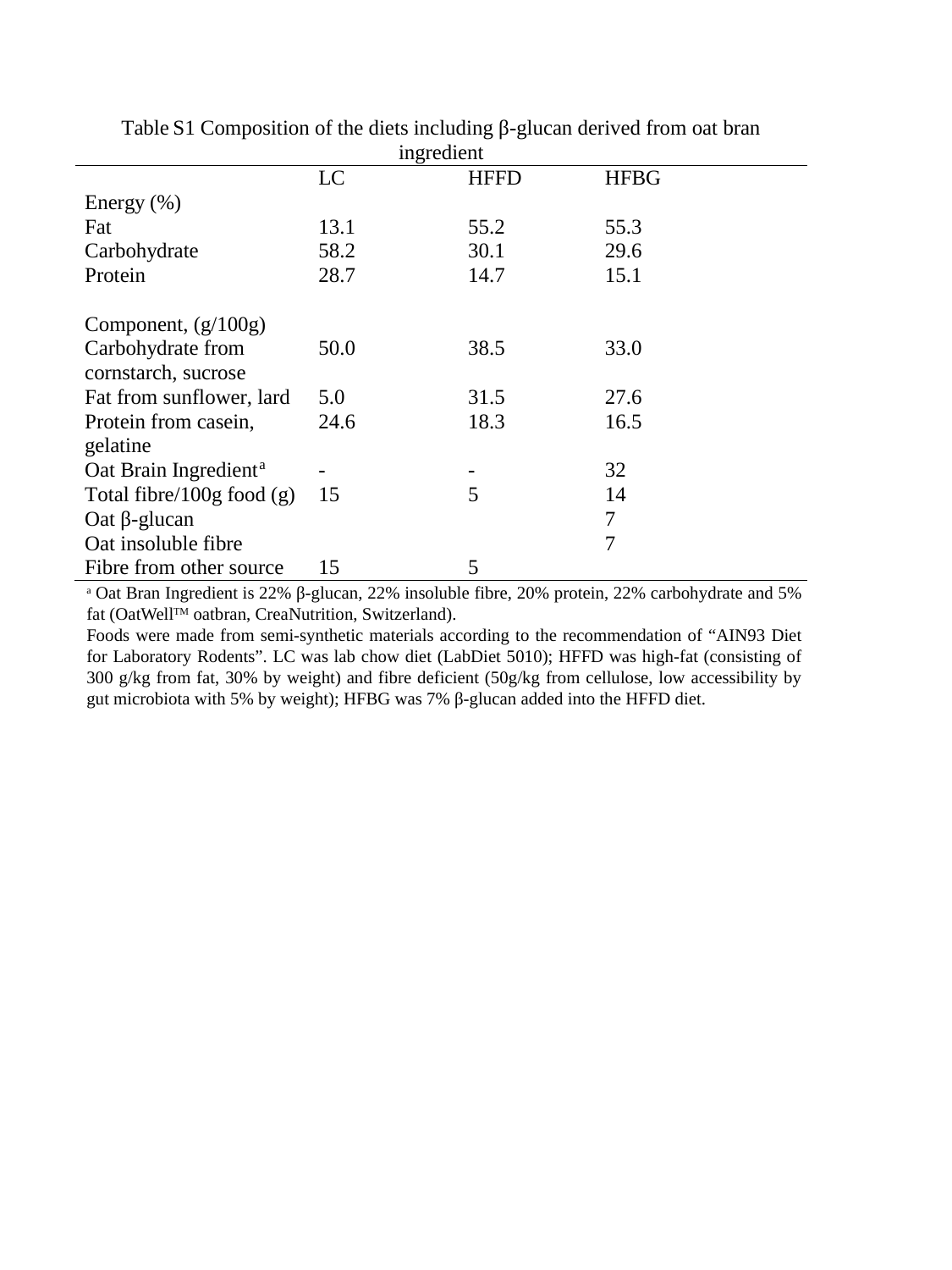

a Oat Bran Ingredient is 22% β-glucan, 22% insoluble fibre, 20% protein, 22% carbohydrate and 5% fat (OatWellTM oatbran, CreaNutrition, Switzerland).
Foods were made from semi-synthetic materials according to the recommendation of “AIN93 Diet for Laboratory Rodents”. LC was lab chow diet (LabDiet 5010); HFFD was high-fat (consisting of 300 g/kg from fat, 30% by weight) and fibre deficient (50g/kg from cellulose, low accessibility by gut microbiota with 5% by weight); HFBG was 7% β-glucan added into the HFFD diet.
