# Supplementary material for: β-glucan attenuates cognitive impairment via the gut-brain axis in diet-induced obese mice
Source: Microbiome. 2020 Oct 2;8:143. doi: 10.1186/s40168-020-00920-y (PMC7532656; doi:10.1186/s40168-020-00920-y)
Supplement: Supplementary file 4 — Additional file 3: Table S2. Predicted KEGG functional pathway differences at level 2 inferred from 16S rRNA gene sequences using PICRUSt after acute HFFD diet with or without β-glucan supplementation. [file 40168_2020_920_MOESM3_ESM.pptx]

## Slide 1
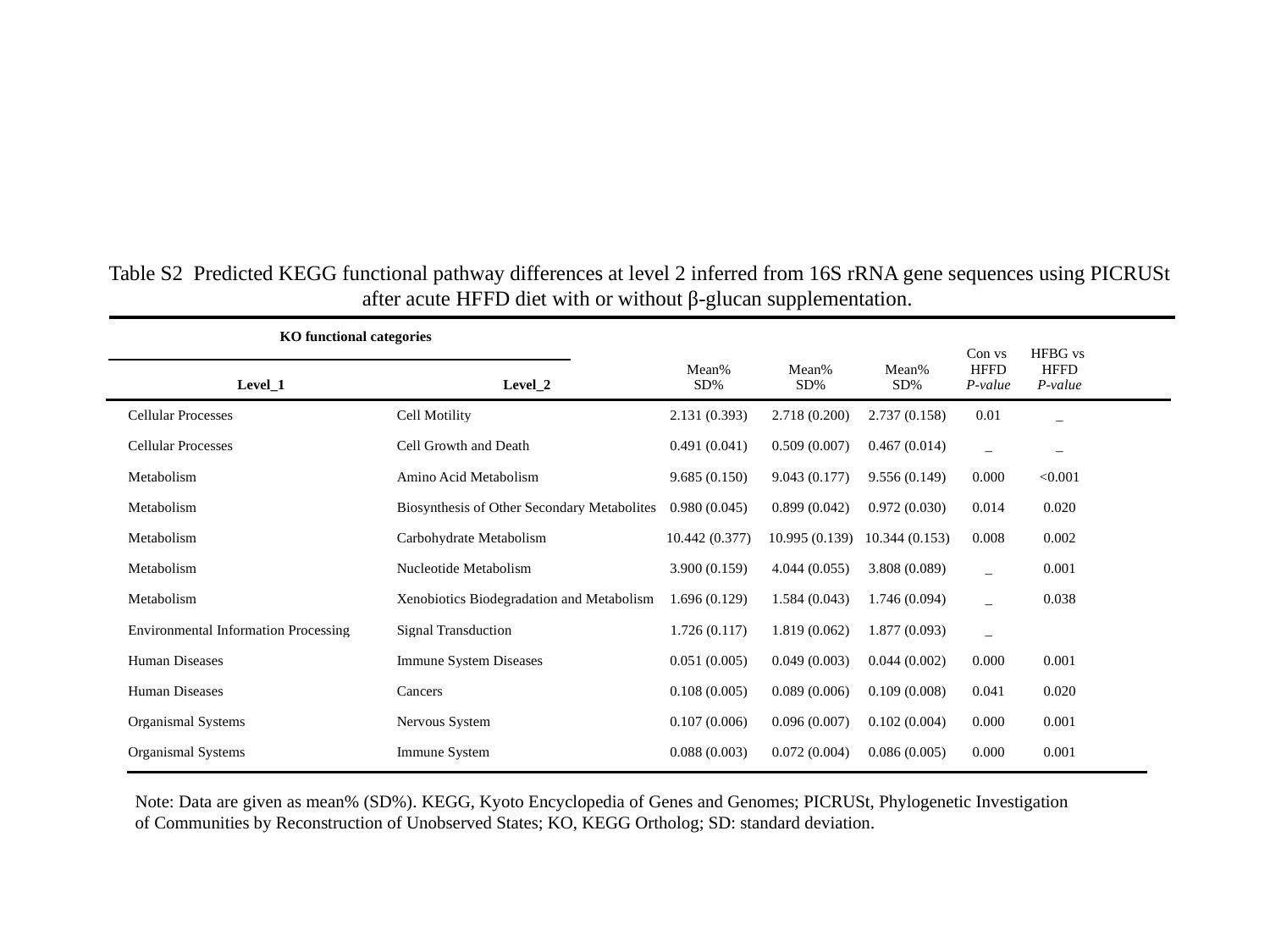

Table S2 Predicted KEGG functional pathway differences at level 2 inferred from 16S rRNA gene sequences using PICRUSt after acute HFFD diet with or without β-glucan supplementation.
KO functional categories
| Level\_1 | Level\_2 | Mean% SD% | Mean% SD% | Mean% SD% | Con vs HFFD P-value | HFBG vs HFFD P-value | |
| --- | --- | --- | --- | --- | --- | --- | --- |
| Cellular Processes | Cell Motility | 2.131 (0.393) | 2.718 (0.200) | 2.737 (0.158) | 0.01 | \_ | |
| Cellular Processes | Cell Growth and Death | 0.491 (0.041) | 0.509 (0.007) | 0.467 (0.014) | \_ | \_ | |
| Metabolism | Amino Acid Metabolism | 9.685 (0.150) | 9.043 (0.177) | 9.556 (0.149) | 0.000 | <0.001 | |
| Metabolism | Biosynthesis of Other Secondary Metabolites | 0.980 (0.045) | 0.899 (0.042) | 0.972 (0.030) | 0.014 | 0.020 | |
| Metabolism | Carbohydrate Metabolism | 10.442 (0.377) | 10.995 (0.139) | 10.344 (0.153) | 0.008 | 0.002 | |
| Metabolism | Nucleotide Metabolism | 3.900 (0.159) | 4.044 (0.055) | 3.808 (0.089) | \_ | 0.001 | |
| Metabolism | Xenobiotics Biodegradation and Metabolism | 1.696 (0.129) | 1.584 (0.043) | 1.746 (0.094) | \_ | 0.038 | |
| Environmental Information Processing | Signal Transduction | 1.726 (0.117) | 1.819 (0.062) | 1.877 (0.093) | \_ | | |
| Human Diseases | Immune System Diseases | 0.051 (0.005) | 0.049 (0.003) | 0.044 (0.002) | 0.000 | 0.001 | |
| Human Diseases | Cancers | 0.108 (0.005) | 0.089 (0.006) | 0.109 (0.008) | 0.041 | 0.020 | |
| Organismal Systems | Nervous System | 0.107 (0.006) | 0.096 (0.007) | 0.102 (0.004) | 0.000 | 0.001 | |
| Organismal Systems | Immune System | 0.088 (0.003) | 0.072 (0.004) | 0.086 (0.005) | 0.000 | 0.001 | |
Note: Data are given as mean% (SD%). KEGG, Kyoto Encyclopedia of Genes and Genomes; PICRUSt, Phylogenetic Investigation of Communities by Reconstruction of Unobserved States; KO, KEGG Ortholog; SD: standard deviation.
