# Supplementary material for: β-glucan attenuates cognitive impairment via the gut-brain axis in diet-induced obese mice
Source: Microbiome. 2020 Oct 2;8:143. doi: 10.1186/s40168-020-00920-y (PMC7532656; doi:10.1186/s40168-020-00920-y)
Supplement: Supplementary file 5 — Additional file 4: Table S3. Pearson correlations between energy intake and metabolic and behavior parameters. [file 40168_2020_920_MOESM4_ESM.pptx]

## Slide 1
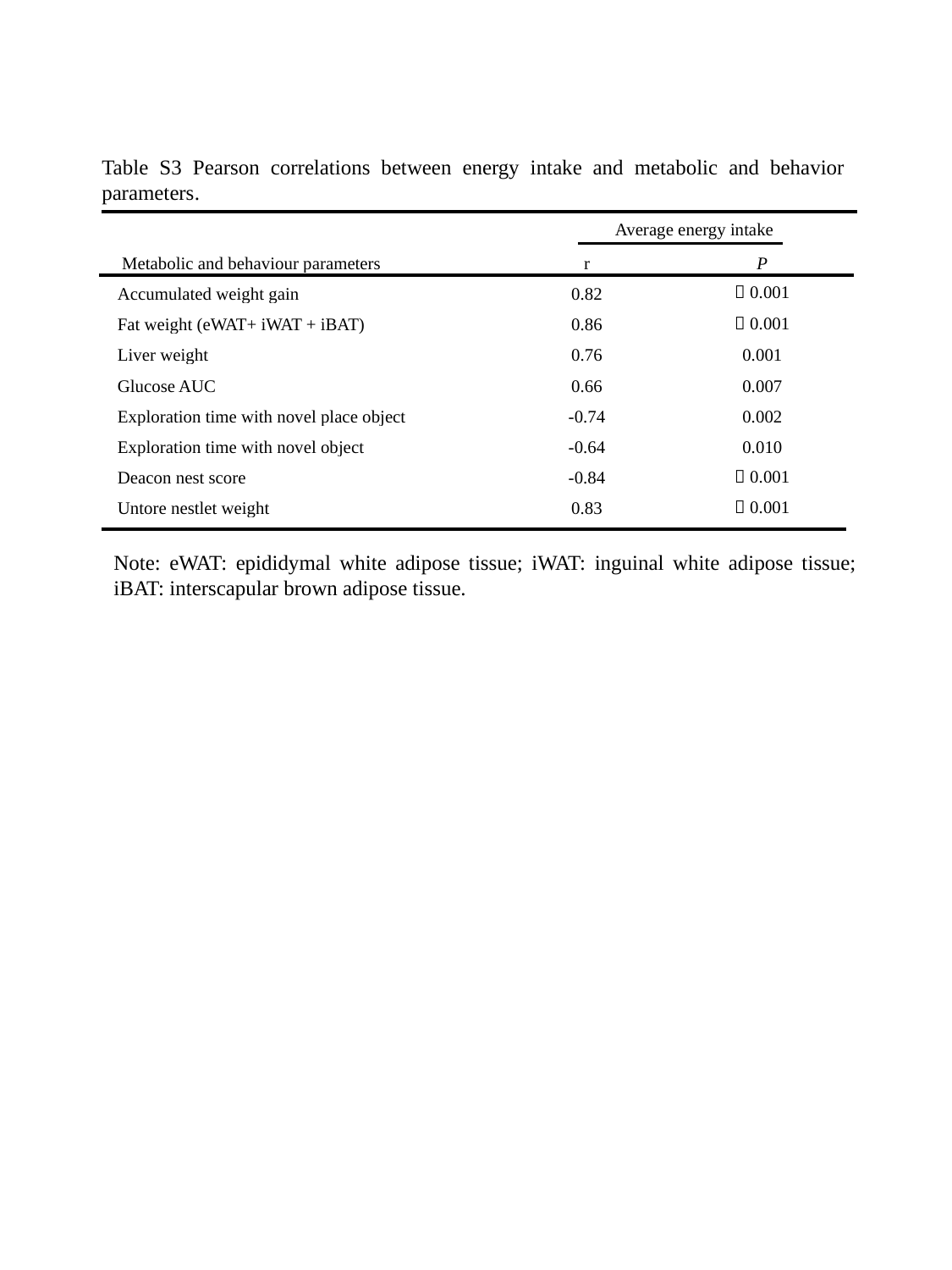

Table S3 Pearson correlations between energy intake and metabolic and behavior parameters.
| Metabolic and behaviour parameters | r | P |
| --- | --- | --- |
| Accumulated weight gain | 0.82 | ＜0.001 |
| Fat weight (eWAT+ iWAT + iBAT) | 0.86 | ＜0.001 |
| Liver weight | 0.76 | 0.001 |
| Glucose AUC | 0.66 | 0.007 |
| Exploration time with novel place object | -0.74 | 0.002 |
| Exploration time with novel object | -0.64 | 0.010 |
| Deacon nest score | -0.84 | ＜0.001 |
| Untore nestlet weight | 0.83 | ＜0.001 |
Average energy intake
Note: eWAT: epididymal white adipose tissue; iWAT: inguinal white adipose tissue; iBAT: interscapular brown adipose tissue.
